# Rational Selection of Minimal Sensor Arrays for Analyte Fingerprinting

**DOI:** 10.1021/acs.analchem.5c07372

**Published:** 2026-04-20

**Authors:** Michael Faran, Gabriel Petresky, Minyeong Yoon, Soo-Yeon Cho, Gili Bisker

**Affiliations:** † School of Biomedical Engineering, Faculty of Engineering, 26745 Tel Aviv University, Tel Aviv 69978, Israel; ‡ School of Chemical Engineering, Sungkyunkwan University, Suwon 16419, Republic of Korea; § The Center for Physics and Chemistry of Living Systems, 35017 Tel Aviv University, Tel Aviv 6997801, Israel; ∥ The Center for Nanoscience and Nanotechnology, Tel Aviv University, Tel Aviv 6997801, Israel; ⊥ The Center for Light-Matter Interaction, 99050 Tel Aviv University, Tel Aviv 6997801, Israel; # The Center for Computational Molecular and Materials Science, Tel Aviv University, Tel Aviv 6997801, Israel; ∇ Sagol School for Neuroscience, Tel Aviv University, Tel Aviv 6997801, Israel

## Abstract

High-dimensional, cross-reactive sensor arrays enable powerful chemical fingerprinting, but practical use is often limited by the difficulty of identifying a small, reliable subset of sensors from screening data that contain only a few replicates per analyte. In many experimental settings, collecting large data sets is infeasible, and sensor selection must be performed under conditions of substantial variability and partial overlap between analyte responses. Here, we present a transparent and data-efficient analysis framework that enables rational reduction of sensor arrays using limited experimental data. Rather than relying solely on black-box accuracy metrics, our approach constructs simple, analyte-specific decision regions from measured responses and evaluates how individual sensors contribute to the separation of analytes that are most difficult to distinguish. Sensors are ranked by their contribution to resolving these difficult cases, producing stable and reproducible selections even when replicate numbers are small. An intuitive trade-off curve directly identifies the smallest sensor subset required to reach a desired classification performance. The robustness of the approach is demonstrated using controlled inflation alteration of experimental variability and by application to three independent fluorescence-based sensor libraries. In all cases, we show that only a few selected sensors are sufficient to achieve low classification error while retaining clear, interpretable decision maps that reveal how sensor responses give rise to analyte discrimination. This work provides an open-source, general, platform-agnostic strategy for screening data analysis and sensor-array design, offering compact and interpretable solutions suited to data-limited conditions common in chemical and biological sensing.

## Graphical abstract

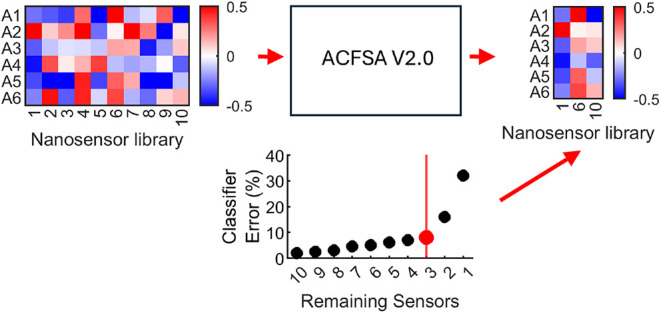

## Introduction

Accurate classification of chemical analytes remains a central challenge in sensor design, particularly when target molecules are diverse, structurally similar, or encountered in complex environments.^1,2^ Traditional sensors often rely on natural molecular recognition elements such as antibodies, enzymes, or aptamers, for one-to-one binding interactions.^3,4^ While highly specific, these systems are typically sensitive to environmental changes, labor-intensive to optimize, and often limited in their applicability across chemically broad analyte libraries.^5^

Rather than relying on selective binding, an alternative strategy is chemical fingerprinting, which uses an array of cross-reactive, semiselective sensors to generate high-dimensional response patterns, namely, a unique “fingerprint” for each analyte.^6–8^ These sensor arrays can be built from diverse platforms (optical, electrochemical, colorimetric, gravimetric, etc.) and have been successfully applied in fields ranging from food analysis and environmental monitoring to medical diagnostics and explosives detection.^9–11^ The resulting complex data sets are then analyzed using statistical tools such as dimensionality reduction, clustering methods, or machine learning models, to enable analyte classification.^12–14^ This approach bypasses the need for highly specific molecular recognition by encoding molecular identity in the collective pattern of responses, offering greater flexibility.

A key requirement for effective fingerprinting is the design of sensor arrays that produce diverse, nonredundant responses across analytes, ensuring each sensor contributes uniquely to the overall fingerprint. Creating such arrays involves carefully selecting sensing elements from a larger pool, often through iterative screening and optimization. This process can be further complicated by sensor drift, signal variability, or batch-to-batch inconsistencies, especially in chemically or biologically complex samples. Moreover, all sensing platforms ultimately require that molecular interactions be transduced into stable and measurable signals. Achieving efficient, reproducible, and real-time signal transduction remains a fundamental challenge in sensor development and may limit performance in practical applications.

Single-walled carbon nanotubes (SWCNTs) provide a uniquely advantageous platform for fingerprinting.^15,16^ Their intrinsic near-infrared fluorescence, photostability, and high surface area make them excellent optical transducers, and their surface chemistry can be modulated in multiple ways to tailor analyte interactions.^17^ A key advantage of SWCNTs is their emission in the near-infrared (NIR) region, particularly between 900–1400 nm, a spectral window where biological samples exhibit minimal autofluorescence, scattering, and absorption.^18–20^ This allows for highly sensitive, low-background detection in complex environments such as biological fluids or tissues.^21–24^ Moreover, the biocompatibility of SWCNTs further extends their utility, enabling detection and monitoring of analytes *in vivo*.^25–34^

SWCNT surfaces can be functionalized noncovalently with peptides, DNA, or polymers, enabling versatile corona phase engineering.^35–38^ Covalent modification, through the controlled introduction of defects, offers an additional layer of tunability.^39–41^ Such defect engineering can modulate SWCNT fluorescence properties and introduce new chemical functionalities at specific sites, further enriching the sensor repertoire.^42–45^ The sensing mechanism of SWCNTs is based on the modulation of their fluorescence by local environmental changes at the nanotube surface.^16,46^ When analytes interact with the SWCNT’s corona phase or exposed defect sites, they can induce changes in polarity, dielectric environment, or charge distribution, leading to shifts in fluorescence intensity or emission wavelength.^47,48^ These optical responses are highly sensitive and occur in real time, enabling SWCNTs to function as dynamic transducers of molecular interactions.^49,50^ Different SWCNT chiralities also expand the sensing landscape by providing distinct emission channels at various wavelengths.^51–56^ By combining corona phase diversity, chirality differences, and both noncovalent and covalent functionalization strategies, one can construct a compact yet information-rich sensor array from a relatively small set of materials.^57–63^

Numerous studies have explored feature- or sensor-subset selection in chemical sensor arrays, where the resulting classifier is termed an electronic nose or e-nose.^64^ Genetic-algorithm wrapper methods have been used to identify compact, high-performing sensor subsets,^65^ and heuristic or exhaustive search strategies have demonstrated strong performance in electronic-nose data sets by directly optimizing classifier accuracy.^66^ Other approaches based on multivariate chemometrics, such as partial least-squares discriminant analysis (PLS-DA) and variable-importance-in-projection (VIP) ranking, have also been widely used to identify informative sensor subsets in electronic-nose and spectroscopy data sets.^67^

Complementary lines of work have introduced mutual-information–based filters^64^ and resolving-power metrics grounded in geometric or information-theoretic principles,^68^ both of which provide elegant criteria for ranking sensors by their discriminative value. Machine-learning approaches, including support vector machines, random forests, and neural-network classifiers, have further broadened the algorithmic toolbox for analyte identification and sensor-array optimization,^69^ and are now widely used to extract patterns from cross-reactive response matrices. Together, these approaches established that reducing the number of sensors can improve robustness, reduce costs, and, in some cases, even enhance classification accuracy.

Nevertheless, the interpretability of results and a good classification performance with limited training data remain challenges in e-noses. Thus, there is a lack of a robust method tailored to sparse experimental screening conditions, where sensor libraries may contain dozens of channels but the number of replicates per analyte is inherently limited, making variance estimation and class separability difficult to quantify reliably. In many biological and chemical sensing applications, the number of available samples per analyte is intrinsically small, often due to limited material, experimental cost, or biological variability, conditions under which standard wrapper and filter methods tend to overfit, produce unstable feature rankings, or fail to generalize.^70^ Consequently, there is a need for sensor-selection frameworks that remain statistically reliable in low-replicate regimes, provide interpretable decision geometry, and explicitly account for uncertainty and class-specific confusion structure rather than relying solely on classifier-driven performance metrics.

In our previous work,^62^ we introduced the Analyte Classification and Feature Selection Algorithm (ACFSA), an unsupervised and interpretable tool designed to identify minimal yet high-performing sensor subsets for analyte classification. ACFSA combines principal component analysis with k-means clustering and a backward feature-elimination strategy, enabling efficient reduction of the sensor set while preserving classification performance. In that study, we applied ACFSA to analyze the fluorescence response patterns of a library of peptide-functionalized, and chemically modified, SWCNT sensors exposed to various metal ions. By systematically eliminating redundant features using a chi-squared metric, ACFSA consistently selected small sensor subsets that yielded generalizable classifiers with high adjusted Rand index (ARI)^71^ values, indicating excellent agreement with the ground-truth analyte classes. This approach enabled accurate classification of five metal-ion analytes across different SWCNT chiralities and oxidation states, establishing ACFSA as a powerful and experimentally relevant tool for optimizing optical fingerprinting sensor arrays under realistic small-sample screening conditions.

Despite these advantages, the original ACFSA framework relied on linear Voronoi decision boundaries in principal-component space, did not explicitly model finite-sample uncertainty in class statistics, and employed a uniform feature-scoring rule that did not account for structured class-pair confusions. As a result, while ACFSA provided a highly effective and interpretable reduction scheme, it did not fully exploit class-specific covariance structure, quantify its overfitting possibility due to sparse experimental screening conditions, nor did it explicitly prioritize sensors that resolve the most difficult class separations. These limitations become increasingly important as sensing problems grow in complexity and class overlap.

In this work, we present a complete and statistically grounded framework for reducing high-dimensional, cross-reactive sensor arrays to compact sensor sets that enable reliable analyte classification under experimentally realistic conditions. The framework, which we refer to as ACFSA V2.0, is designed for screening scenarios in which replicate numbers are limited, each analyte sample is assumed to be drawn from a Gaussian distribution, and analyte response distributions partially overlap. Under such conditions, many existing sensor-selection strategies become unstable or difficult to interpret. Rather than relying on purely linear partitioning or empirical accuracy metrics, the approach models analyte responses using class-specific statistical descriptions that explicitly account for experimental variability, enabling curved decision boundaries that better reflect measured data. Sensor selection is guided by how effectively individual sensors contribute to separating analytes that are hardest to distinguish, rather than by overall classification performance alone. In parallel, we introduce a principled procedure for identifying an operating point that balances classification accuracy against sensor count, providing a transparent criterion for selecting minimal yet robust sensor subsets. Beyond methodological development, we evaluate the framework using controlled perturbations of experimental variability and demonstrate its generality across three independent fluorescence-based sensor libraries: 30 peptide-functionalized SWCNT sensors fingerprinting 5 metal ions,^62^ 20 DNA-SWCNT sensors distinguishing 3 sweat-related analytes,^72^ and an additional library of 10 DNA-SWCNT sensors screened against 6 common analytes found in urine.^45^ Together, these results establish a modular and interpretable pipeline for sensor-array reduction and classification that is transferable across sensing platforms and readout modalities. To facilitate transparency, reproducibility, and adoption by the broader analytical community, we provide a fully open-source implementation of the framework. While demonstrated here using near-infrared fluorescent SWCNT fingerprints, the framework is broadly applicable to cross-reactive sensor arrays and directly addresses the challenges posed by limited data, per class-Gaussian behavior, and the need for physical interpretability in chemical and biological sensing.

## Methods

### Experimental Data Sets

The data sets analyzed in this work originate from previously published screening experiments of near-infrared fluorescent SWCNT nanosensor libraries exposed to multiple analytes. These data sets include: (i) a library of peptide-functionalized SWCNT sensors screened against five transition metal ions, (ii) a DNA-SWCNT nanosensor library for sweat analyte detection, and (iii) a DNA-SWCNT nanosensor library for urinary analytes. The preparation, characterization, and fluorescence screening procedures for these nanosensor libraries are described in detail in the original studies.^45,62,72^ A short summary of the data sets used here is provided in Supporting Information (SI) Section S1.

### ACFSA V2.0 Scheme

#### Scope and Novelty of ACFSA V2.0

The prior version of AFSCA^62^ used an unsupervised k-means mode^73^ with posthoc Hungarian-algorithm matching.^74^ In contrast, we introduce *ACFSA V2.0*, a supervised workflow aligned with screening settings where analyte classes are known *a priori*. The new ACFSA V2.0 includes (i) finite-sample covariance inflation and regularization when estimating the per-class Gaussians in the PC1–PC2 plane obtained by principal component analysis (PCA).^74^ This is implemented using shrinkage toward a pooled covariance target in using the Ledoit–Wolf regularization,^75^ combined with a small ridge/eigenvalue floor to ensure numerical stability of the covariance matrices.^76^ (ii) QDA (quadratic discriminant analysis) decision regions^77^ with the per-class covariance regularization by default, with Voronoi^78^ as fast alternatives; (iii) a weighted chi-squared feature-selection rule alongside a default unweighted uniform baseline,^79^ where the samples labels inform both binning scheme; and (iv) a cost function to select the sensor-count vs error working point. We next present the end-to-end pipeline, followed by brief notes on mean error calculation and each change, further supporting pseudocode, analytical derivation, computational details, and runtime profiling appear in the SI.

#### Pipeline Overview

ACFSA V2.0 seeks the smallest sensor subset that preserves class separability, without a dedicated hold-out set. Given $X\in R^{n\times p}$ (samples × sensors) and labels *y* ∈ {1,···,*K*}^*n*^, we (i) compute per-sensor fractional responses and subtract the mean from the corresponding data points to mean-center each sensor data; (ii) compute a global PCA for inspection and visualization (PC1 and PC2 are retained, and sample scores are shown in that space), evaluated on the current sensor set; A cumulative explained variance flag is added as a hyperparameter option: If PC1 explains more than 95% of the variance (hyperparameter), the scheme switches to a one-dimensional decision model using PC1 only, applying the same QDA or Voronoi logic; unless stated otherwise, this flag is not activated to preserve the two-dimensional visualization of decision boundaries. (iii) Within each class, we mean-center the class PC1/PC2 scores, estimate the 2 × 2 covariance with finite-sample inflation, perform a class-specific PCA on the centered scores to obtain the ellipse axes, and plot the corresponding 95% confidence ellipse in the global PCA plane. (iv) train a classifier (QDA by default; Voronoi as fast, smooth-boundary alternatives); and (v) record the per-class mean estimated classification error, ARI, and mean separation ⟨*D*⟩, defined as the distance between the class centroids, as functions of the current number of sensors. (vi) We then run feature selection by removing one sensor per iteration using either chi-squared or weighted chi-squared (wFS; pairwise χ^2^ scores weighted by the inverse interclass distance, sep_*ab*_, defined between class *a* and *b* samples centroids in the decision lines diagram). The loop continues until a single sensor remains; the resulting traces allow choosing a working point, defined by sensor count vs error of the scheme. We emphasize that, for the estimated classification error, the class-specific covariance matrices are assumed to be adequately estimated, which is only a coarse approximation from small data set^75^ used to select a working point for the classifier with respect to the cost function, since covariance estimation from limited sample sizes is inherently challenging.^75^

This pipeline is presented in sections S2 and S3 of the SI: Table S1 denotes the pipeline parameters, while Algorithm S1 and its utility functions (Algorithms S2, S3, and S4) are presented afterward.

### Per-Class Error Estimation

For each class, a 2D Gaussian distribution is fitted to its samples in the PC1-PC2 space. Using the current decision diagram (Voronoi or QDA), the spatial region assigned to that class is identified. The overlap between the fitted Gaussian and its own decision region is then estimated numerically. This is done by generating a 2D grid with sufficient spatial resolution across the PC space. The region boundaries are defined according to the decision diagram, and random samples are drawn from the class’ Gaussian. The proportion of these simulated data points that fall inside the class’s own region is computed by dividing the number of points within the boundaries by the total number of simulated points. This fraction represents the integral of the class Gaussian over its assigned region. This AFSCA V2.0 error calculation is based on assuming Gaussianity of each class in the PC1–PC2 space^62^ and calculating its overlap with other classes’ decision regions, instead of using a holdout set of labels for validation. The analyte classes Gaussianity in the PC space stems from the sensor measurements Gaussianity assumption, as the classes are a linear transformation of the original sensor data. We emphasize that if the PC1 explains more than 95% of the variance and the cumulative explained variance flag is activated, this process is conducted on a 1D grid instead, keeping the same decision lines logic in 1D.

### Accounting for Experimental Statistical Uncertainty

As mentioned before, class distributions remain Gaussian in the PC1-PC2 space. Nevertheless, experimental screening data sets often contain very few replicates per class, defined as *n*_*k*_ (*n*_*k*_ ⩽ 5 for class *k*), so estimating class moments from limited data incurs substantial uncertainty.^80^ In particular, naïve plug-in estimates for each class mean and variance understate predictive variability. To address this, we inflate, perform shrinkage and perform eigenvalue conditioning for each class covariance matrix to reflect finite-sample uncertainty in both the measured mean and covariance matrix. We emphasize that if the PC1 explains more than 95% of the variance and the cumulative explained variance flag is activated, this process is conducted on the sole 1D variance instead of the covariance matrix.

#### Predictive Variance Inflation

For each class *k*, we compute a local PCA using only the samples belonging to that class. In general, these local PC1–PC2 axes differ from the global PCA computed over all samples, effectively rotating the class geometry relative to the global coordinate system.

Consider a new observation drawn from class *k* along one of its local PCA axes, where the class mean and variance along that axis are unknown and denoted by (μ_*k*_, σ_*k*_^2^). Given *n*_*k*_ samples in class *k*, the posterior predictive variance for this draw is (see Section S3 in the SI for derivation):

1 $${\mathrm{\sigma ̃}}_{k,\mathrm{pred}}^{2}=s_{k}^{2}\underset{\mathrm{unknown}\;\mathrm{mean}}{\underset{︸}{\left(1+\frac{1}{n_{k}\;}\right)}}\times \underset{\mathrm{unknown}\;\mathrm{variance}}{\underset{︸}{\frac{n_{k}\;-1}{n_{k}\;-2}}}\left(n_{k}>2\right)$$

where *s*_*k*_^2^ is the unbiased sample variance along the corresponding local PCA axis. This expression accounts for uncertainty in both the estimated mean and variance. We use σ̃_*k*_^2^ ≡ σ̃_*k*,pred_^2^ to construct a diagonal covariance matrix in the local PCA coordinates, which is then rotated back to the global PC1–PC2 space to obtain the inflated class covariance used by the chosen classifier (e.g., QDA) and for synthetic resampling. Thus, predictive variance inflation enlarges the class Gaussian along its principal directions while preserving its orientation.

We assume at least two samples per class. Predictive variance inflation accounts for uncertainty in the estimated mean and variance in the per-class PC1–PC2 coordinates; however, if all class covariances are inflated by the same scalar factor, quadratic discriminant analysis (QDA) decision boundaries remain unchanged.^77^ Moreover, off-diagonal covariance elements are particularly sensitive to small sample sizes,^75^ leading to unstable estimates. Therefore, variance inflation alone corrects uncertainty estimates but does not stabilize classifier geometry in finite-sample regimes, motivating additional covariance regularization. Class covariance matrices in the PC1–PC2 subspace were regularized to ensure stable QDA decision boundaries under small-sample conditions. The procedure combines pooled-covariance shrinkage^77^ with eigenvalue conditioning, with shrinkage weights estimated using a Ledoit–Wolf–style approach.^75,76^ The full derivation and implementation details are provided in Section S3 of the SI.

### Voronoi Diagram and QDA

In the initial version of ACFSA, we used the Voronoi decision lines to obtain piecewise-linear boundaries and a low computational cost (single pooled covariance). However, when classes exhibit unequal covariances or curved boundaries, QDA typically improves separability by considering the covariance matrix per class. Unstable covariance matrix estimates may degrade QDA classification accuracy; accounting for experimental statistical uncertainty can mitigate this effect, as described above. ACFSA V2.0 therefore adopts QDA as the default while retaining Voronoi as an option when boundaries are approximately linear or speed/simplicity is preferred.^81^

### Feature Selection Methods

For each sensor and each class, we compute the per-class mean and variance from all samples sharing that label. If 1D–QDA binning is selected instead of Voronoi binning, the per-class variances for each sensor are first inflated in a manner analogous to eq 1, and then regularized using pooled-covariance shrinkage with eigenvalue conditioning adapted to the one-dimensional case (see SI Section S3: 1D QDA Binning for Feature Selection).

Next, the sensor data is partitioned into class-specific groups based on their labels, each group is tagged with its mean, and the readings are reordered so that the groups appear in increasing mean order (lowest first, highest last). Using the precomputed per-class marginals, we derive nonuniform thresholds from the chosen classifier’s pairwise decision boundaries—Voronoi midpoints between the class means when using Voronoi, or the 1D QDA computed thresholds otherwise. The distinct thresholds define a sensor-specific “ruler”: between any two consecutive thresholds, the classifier assigns a specific class to any value in that interval. Using this ruler yields nonuniform, decision-aware binning is performed. We then discretize all sensor readings by mapping each value to its class via the ruler. Compared with uniform binning, this scheme leverages label information and the optimal separation lines implied by the (approximately Gaussian) per-class distributions, concentrating resolution near true decision boundaries and improving sensitivity to confusable classes.

Using the above sensor discretization, we choose one of two schemes: uniform feature selection (uFS) or weighted (wFS), each with its own scoring, where uFS is defined as the default choice of the algorithm. In uFS, we rank sensors by Pearson’s χ^2^ between the sensor’s discretized bins and the ground-truth label vector (the bin × label contingency), reflecting the sensor’s ability to classify samples on its own. In wFS, for every class pair (*a*,*b*) we recompute Pearson’s χ^2^ on the restriction to *a*, *b* (using the same bins) and then average the pairwise statistics with weights *w*(*a*, *b*) ∝ 1/Δ­(*a*, *b*), ∑_*a*<*b*_ *w*(*a*, *b*) = 1, where Δ­(*a*, *b*) measures current separability as the distance, along the sensor axis, between the centroids of the two class *zones* induced by the chosen decision model (QDA or Voronoi). Boundaries, and thus bins, are recomputed at each iteration so the discretization remains aligned with the evolving classifier. The overall time complexity calculation of AFSCA V2.0 appears in section S4 of the SI.

### Error–Sensor Count Working Point Selection

The operating point is the final subset of sensors chosen by the experimentalist after reviewing ACFSA V2.0 performance across iterations. Let *e*(*l*) denote the mean classification error after selecting *l* sensors. We choose the operating point by minimizing a cost that trades accuracy for parsimony:

2 J(l)=e(l)+ηl

where η ≥ 0 is the cost penalty increase *per additional sensor*. This cost function aims to balance the common trade-off faced by the experimentalist, where more sensors yield greater accuracy but require additional labor. Given η, we select

3 $$l^{*}=\arg\min_{l\in 1,...,p}J\left(l\right)$$

and report the full *e*(*l*), ARI­(*l*), and *D*(*l*) curves together with *l* ^*^. In practice, we use two η values: 3% (default) and 0.5% (alternative).These parameter choices were hypothesized to reflect low and high penalty for additional sensors, where the latter prioritizes accuracy over sparsity when a slightly larger subset is acceptable. This hyopthesis is tested later on. In applications, η can be tuned using domain knowledge.

## Results and Discussion

### Demonstration of ACFSA V2.0 on a Representative Data Set

#### The Default ACFSA V2.0 Activation

To demonstrate the ACFSA V2.0 workflow, we applied it to a representative data set comprising fluorescence responses from a library of SWCNT-based nanosensors exposed to metal-ion analytes.^62^ The data set is summarized in Figure 1 as a heat map showing the mean normalized fluorescence response across sensor–analyte pairs. Rows correspond to analytes and columns to individual sensor library members (1–30), each defined by its chirality, peptide corona, and its oxidation state (sensor identifiers listed in Table S2 of the SI). The cell color encodes the averaged response across five replicates per analyte.

**Figure 1:**
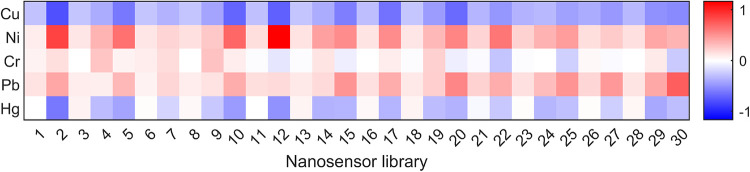
Screening heat map of SWCNT-nanosensor fluorescence responses to metal ions. Rows correspond to Cu, Ni, Cr, Pb, and Hg analytes; columns enumerate the nanosensor SWCNT library (*p* = 30). The corresponding sensor labels are listed in Table S2 of the SI. Colors indicate the normalized response, highlighting class-dependent fingerprints across sensors. Data reproduced with permission from Petresky et al.^62^

Following the ACFSA V2.0 workflow (Figure 2A), we applied a global PCA to the entire data set (Figure 1), yielding principal components that capture weighted linear combinations of the sensor response vectors. The first two components, ranked by their latent values, explained 89.3 and 4.6% of the total variance, respectively, before sensor elimination, and were used for subsequent analysis. Using the known analyte labels, we grouped measurements by class and fitted Gaussian distributions in the two-dimensional PC space, preceded by local PCA within each class subset. To account for limited replicates (*n*_*k*_ = 5 per class), covariance estimates were inflated according to eq 1, as illustrated schematically in Figure 2B. Each class was represented by a 95% confidence ellipse in PC space. From these class-specific Gaussian means and variances, QDA decision regions were derived, replacing the Voronoi boundaries used previously (Figure 2C).

**Figure 2:**
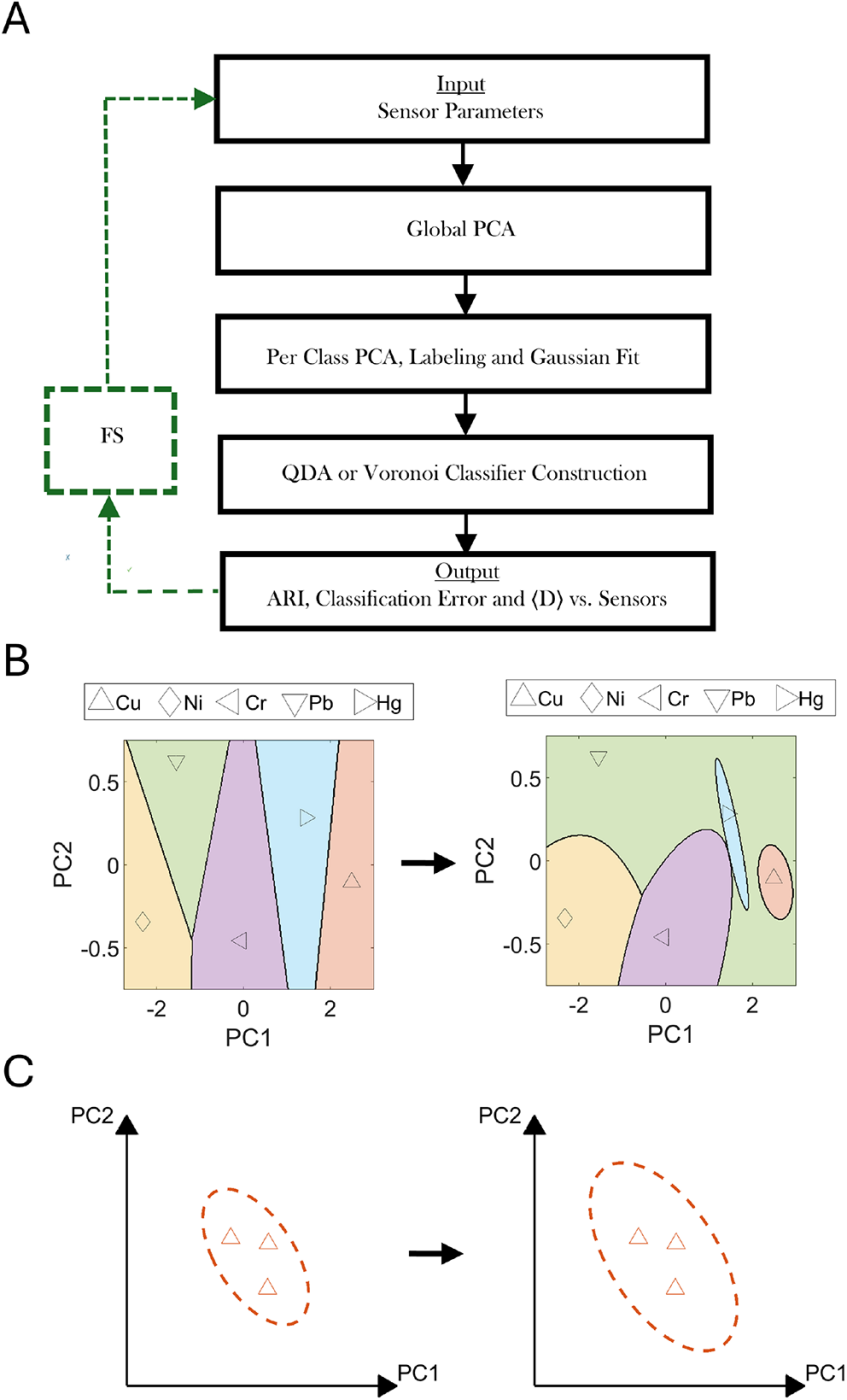
Overview of the updated ACFSA V2.0 workflow and refinements. (A) ACFSA V2.0 scheme: different steps are marked in black according to their execution order; the green box marks the feature-selection (FS) feedback loop. (B) The transition of decision boundaries in the PC1–PC2 plane from Voronoi (left) to QDA (right), enabling curved boundaries that better handle overlapping classes. (C) Necessary variance inflation, shrinkage toward the pooled covariance and ridge regularization are applied to each class Gaussian in PC1–PC2 space to account for estimation error with few samples per class (small *n*), yielding more conservative class 95% confidence ellipses.

Results from the first iteration are shown in Figure 3A, with uncertainty ellipses and latent PCA values for the first two components, appearing on the axes labels (Figure 3A, left column) and QDA zones (Figure 3A, middle column) in the upper panels. The adjusted Rand index (ARI), mean interzone distance, and classification error were computed and stored at each iteration (Figure 3A, right column). Feature elimination proceeded iteratively using the unweighted, univariate chi-squared selection rule, repeating the classification cycle until only one sensor remained. Two working points were defined from the trade-off between mean classification error and sensor count, namely, the default and an alternative, as shown in Figure 3A, right column. To assess whether the two working points, defined by the penalty increase η in eq 3, correspond to distinct regimes for this data set, we performed a sensitivity analysis of the selected number of sensors as a function of η (Figure S1 in the SI). We found that the two chosen operating points lie within different stable regions of this curve. We emphasize that this behavior is data set-specific; however, these values can serve as reasonable initial baseline choices, which may be adjusted based on domain knowledge and experimental constraints for other data sets. For the two last remaining sensors in the scheme, the fitted confidence ellipses and corresponding PCA latent values on the axes labels and QDA decision map appear in the lower panels of Figures 3A (left column) and Figure 3A (middle column), with the sequence of sensor eliminations detailed in Table S3. Overall, the scheme achieved ∼2% classification error with one selected sensor, which decreased to 0.01% upon adding a second sensor. These results demonstrate that in this data set, the number of sensors required can be drastically reduced while maintaining low classification error. Moreover, adding additional sensors does not necessarily improve performance and may even degrade it by introducing noise rather than an informative signal, as illustrated in Figure 3A (right column).

**Figure 3:**
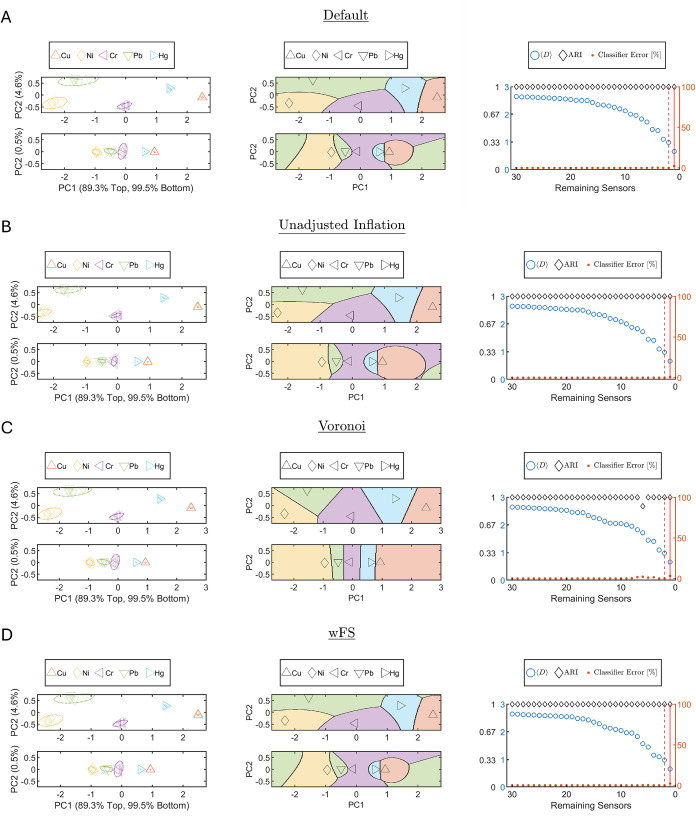
Overview of ACFSA V2.0 activation variants and performance across parameter settings. (A) Default activation parameters. (B) Unadjusted Inflation. (C) Voronoi tessellation. (D) Weighted feature selection (wFS). Left column: Geometry in the PCA plane, 95% confidence ellipses for each class overlaid on sample points (colors/markers per legend) in PC1–PC2 space. The top subpanel shows the first ACFSA V2.0 iteration result; the bottom shows the result of the last two remaining sensors. Middle column: Decision regions derived from the fitted class Gaussians (QDA for (A), (B), and (D); Voronoi for (C)) for the first iteration (top) and the last two remaining sensors (bottom). Black markers indicate the Gaussian means used to define each region; region colors match those in the left column. Right column: Progress of performance vs subset size: ⟨*D*⟩ and ARI (left axis) and mean classification error [%] (right axis) as functions of the number of remaining sensors, with markers matching the legend. The vertical solid red line marks the default working-point sensor subset; the vertical dashed red line marks the alternative working point (see main text). For each variant, the list of the eliminated sensors in each iteration appears in Tables S3–S6 of the SI.

#### Alternative ACFSA V2.0 Activation Modes

We ran ACFSA V2.0 on the same data set with three variants: (i) unadjusted inflation i.e., ignoring estimation uncertainty (Figure 3B), (ii) Voronoi tessellation in place of QDA (Figure 3C), and (iii) weighted rather than uniform feature selection (Figure 3D). appears in SI Tables S4, S5, and S6, respectively.

Across all variants, the per-class error profiles (Figure 3 right column) retained a similar shape, and the working points (sensor counts and identities for each working point selection) were unchanged (see SI Tables S4, S5 and S6), indicating robustness of the sensor choice for this examined data set. The Voronoi variant used in our previous ACFSA version^62^ without the automated label-matching, yielded a slightly higher error with one sensor (from 2% to 3.1%), consistent with its suboptimality for Gaussian classes relative to QDA. The unadjusted inflation case produced lower apparent error due to smaller class-uncertainty estimates (from 2 to 0.8% in the last remaining sensor, for example); however, without priors on the mean and variance, this underestimates generalization error and should be interpreted only as a sanity check confirming that reduced Gaussian uncertainty improves classification. Nevertheless, the unadjusted inflation case for one sensor can be compared to our previous work.^62^ Using all the sensors, ACFSA V1.0 yielded the sensor (6,5)–*SWCNT*-*Gly*_NOX_ (sensor 5 in Table S2) as the last remaining sensor in the elimination scheme, with a mean classification error of 3.1%. In comparison, the unadjusted inflation ACFSA V2.0 activation yielded the same last remaining sensor (see Table S4), with a mean classification error of 0.8%. The sole difference between the two is the 1-D separation lines between classes, where QDA is conducted for the latter. This highlights the superiority of QDA for class separation under the assumption of Gaussian class distributions, compared to the Voronoi (nearest-centroid) boundaries.The weighted feature selection, displayed in Figure 3D. achieved a similar classification error profile to the default feature selection Figure 3A. with negligible differences. For example, the feature selection was identical for the last four iterations, and thus the QDA maps are identical with the same classification error for both cases. Less than 0.1% differences in estimated classification error then exists due to different feature selection for other iterations, which indicates a negligible difference for this data set.

### Application of ACFSA V2.0 to Artificial Data Sets

To evaluate the sensitivity of ACFSA V2.0 to measurement uncertainty, we constructed artificial variants of the metal-ion data set. Using the default activation mode as a baseline (Figure 3A), we extracted the per-class mean and variance values from the first ACFSA V2.0 iteration in the PC space. Since PCA is a linear transformation of Gaussian-distributed sensor data, inflating the variance in the PC space is equivalent to resampling each sensor’s Gaussian distribution with enlarged variance.

We applied standard deviation (STD) inflation factors of 2, 5, and 10, multiplying the baseline uncertainty inflation by these factors. Apart from this modification, all activation parameters were kept identical to the default. For each class, 50 synthetic Gaussian samples were generated with unchanged means but inflated standard deviations. We assumed that global and local PCA loadings remained unchanged, with only the covariance matrices scaled by the square of the chosen factor. These synthetic samples were used to redraw class uncertainty ellipses and rerun the full ACFSA V2.0 feature-selection loop until only a single sensor remained. Results are shown in Figure 4, ordered analogously to the default case, where Figure 4A,B,C correspond to inflation factors of 2, 5, and 10, respectively. The corresponding sensor elimination orders appear in SI Tables S7, S8, and S9, respectively.

**Figure 4:**
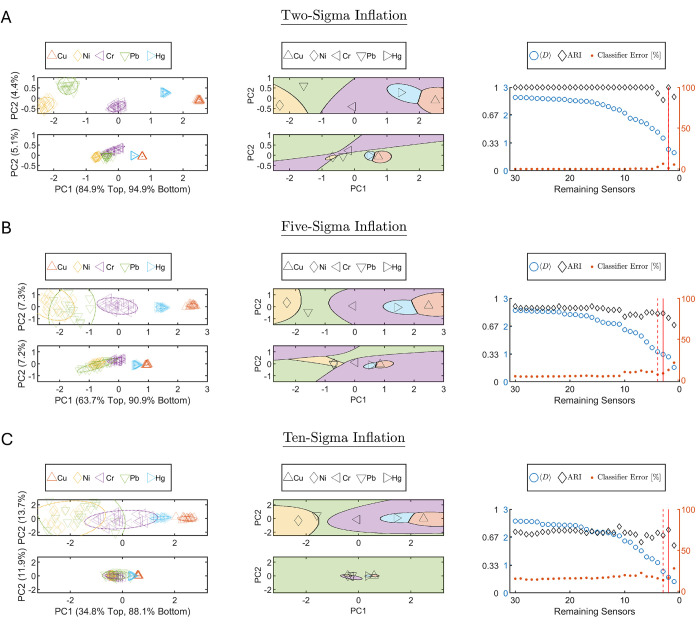
Sensitivity analysis to controlled class overlap using synthetic Gaussian resampling. Data are resampled from the baseline class Gaussians with each default class standard deviation scaled by (A) × 2-sigma, (B) × 5-sigma, and (C) × 10-sigma. Note that default Gaussians already include the small-sample inflation. Left column: Geometry in the PCA plane 95% confidence ellipses with sample points (colors/markers per legend) in PC1,PC2 space; the top subpanel shows the first ACFSA V2.0 iteration and the bottom the selected working point. Middle column: QDA decision regions for the same two iterations; black markers indicate Gaussian means, and region colors match the left column. Right column: Performance versus remaining sensors: ⟨*D*⟩ and ARI (left axis) and mean classification error [%] (right axis). The vertical solid red line marks the default working-point sensor subset; the vertical dashed red line marks the alternative working point (see main text).

Results from the first and last iterations for each inflation level appear in Figure 4, with uncertainty ellipses (Figure 4, left column) and QDA zones (Figure 4, middle column) in the upper and lower panels, respectively. The PCA projections in the left column of Figure 4 compare the first-iteration topology (top subpanels) with the final two-sensor configuration (bottom subpanels) under different covariance inflation levels. For two-sigma inflation (panel A), PC1 (PC2) explains 84.9% (4.4%) of the variance in the first iteration and 94.9% (5.1%) in the final configuration. For five-sigma inflation (panel B), PC1 (PC2) explains 63.7% (7.3%) and 90.9% (7.2%), respectively. For ten-sigma inflation (panel C), the corresponding PC1 (PC2) values are 34.8% (11.9%) and 88.1% (13.7%). As the assumed classes’ variance increases, the effective structure in the PC1–PC2 plane becomes increasingly compressed, suggesting that the two-dimensional representation may limit separability under higher uncertainty assumptions. This observation indicates that extending the framework to higher-dimensional decision spaces may further improve performance, an avenue for future work.

The per-class error exhibits an increasing trend with the number of surviving sensors, while the ARI decreases (Figure 4, right column). This result is consistent with the intuition that fewer sensors cause less accuracy. Moreover, increasing inflation consistently reduces ARI and increases mean classification error, as expected. Across all inflation level, we observe an overarching trend in which per-class error decreases and ARI increases as the number of sensors grows. Although the dependence is not strictly monotonic, the tendency is evident, reflecting the intuition that adding sensors generally improves performance in difficult classification tasks, though not invariably. At the default working point, the error rose from 0.9 to 8.5% and 16.5% for inflation factors of 2, 5, and 10, respectively. These trends are also evident in the QDA decision boundaries (Figure 4, middle column), where inflated uncertainty changes interclass boundaries. Notably, for the 10× inflation case (Figure 4C middle column), most of the decision map collapses to predicting Pb as the analyte green color in the decision map, reflecting the dominance of its inflated uncertainty. The default scheme retained 2, 3, and 2 sensors, for inflation factors 2, 5, and 10, respectively, while the alternative scheme retained 2, 4, and 3 sensors, respectively. The identities of the retained sensors likewise changed for each case, as detailed by the corresponding labels in SI Tables S7, S8, and S9 (the last remaining sensors are eliminated last, described by the bottom rows).

Overall, these results confirm that ACFSA V2.0 is sensitive to the degree of statistical uncertainty in the data, with larger inflation leading to degraded clustering and classification performance. This underscores the necessity of principled uncertainty inflation in experimental data sets to avoid optimistic bias and to obtain reliable operating points.

### ACFSA V2.0 Benchmarking

Benchmarking of ACFSA V2.0 against several widely used wrapper-based feature-selection approaches, including SVM-RFE, RF-RFE, and PLS-DA-VIP-RFE,^82^ using a fully nested leave-one-out cross-validation (LOOCV) framework^83^ indicates that the ACFSA framework achieves competitive or improved classification accuracy in the small-sensor regime while remaining computationally efficient. Detailed benchmarking results, including comparisons with ACFSA V1.0, runtime analysis, and synthetic downsampling experiments, are provided in SI Section 7, including Figure S2 and Tables S10–S14.

### Cross-System Demonstration of ACFSA V2.0

To evaluate the generalizability of ACFSA V2.0 beyond the original system, we selected two additional representative data sets as case studies: Lee et al.^72^ (data set 1) and Yoon et al.^45^ (data set 2). Both employ NIR fluorescent SWCNT sensor arrays but differ in sensing configuration and analytical purpose, providing complementary tests for evaluating ACFSA V2.0 for different systems. Data set 1 comprises *p* = 20 sensors of SWCNTs functionalized by various DNA sequences, screened against an analyte library of Folate (Fol), Glucose (Glc), and Urea (Ure),^72^ whereas Data set 2 includes *p* = 10 SWCNT sensors screened against Creatinine (Cr), Glucose (Glc), Lactate (Lac), Tyrosine (Tyr), Urea (Urea), and Uric acid (UA).^45^ Both data sets possess *n*_*k*_ = 3 samples per class. Screening heat maps of normalized fluorescence responses are shown in SI Figures S3 and S4, respectively. Sensor labels are listed in SI Tables S15 and S16, respectively. Their elimination order is listed in SI Tables S17 and S18 correspondingly. These studies serve as independent examples for assessing how ACFSA V2.0 performs across distinct sensor sets and analyte libraries.

For data set 1, we employed the default activation of ACFSA V2.0 with the exception of using weighted feature selection (wFS) instead of uniform selection. This choice was motivated by the wFS objective of maintaining separation between closely related classes throughout the algorithm iterations, even at the potential cost of reduced accuracy for more distant classes (see Methods). The resulting analysis is shown in Figure 5A, following the same structure as earlier figures. Clearly, the Fol class is separated from the two closer classes (Glc and Ure), illustrating the rationale for using wFS in this case (Figure 5A, left and middle columns). The first iteration two principal components accounted for 96.3 and 2.0% of the total variance, respectively. Additionally, the remaining two sensors two principal components accounted for 97.3 and 2.7% of the total variance, respectively.

**Figure 5:**
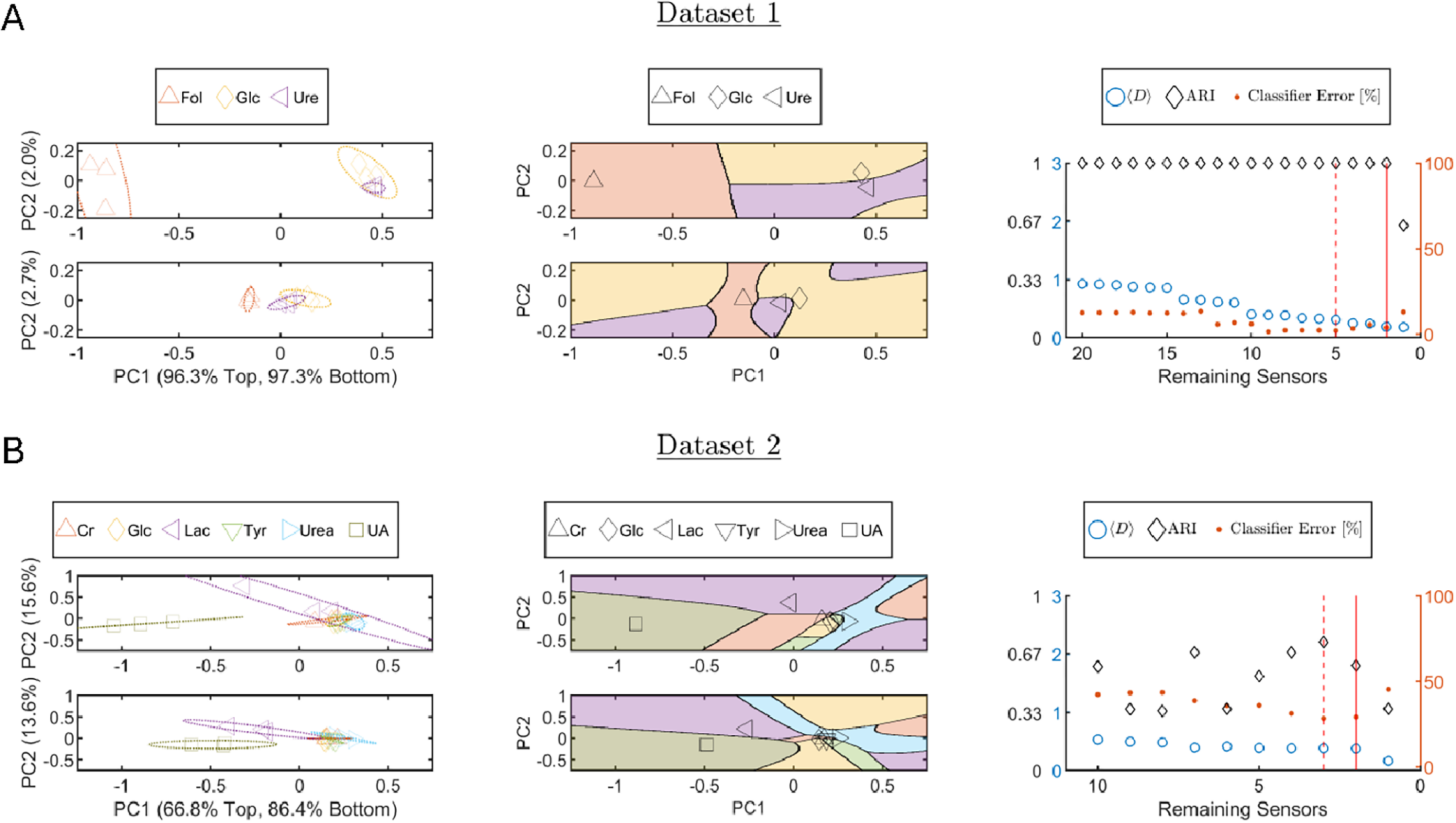
Cross-system application of ACFSA V2.0, with the goal of analyte classification. (A) Data set 1, used with permission from Lee et al.,^72^ evaluated with ACFSA’s default activation using weighted feature selection (wFS). Sensor elimination order appears in SI Table S17. (B) Data set 2, used with permission from Yoon et al.,^45^ under the default activation. Sensor elimination order appears in SI Table S18. Left column: Geometry in the PCA plane, 95% confidence ellipses overlaid on sample points (colors/markers per legend) in PC1, PC2 space; the top subpanel is the first ACFSA V2.0 iteration and the bottom the selected working point. Middle column: QDA decision regions for the same two iterations; black markers denote Gaussian means and region colors match the left column. Right column: Performance vs remaining sensors: ⟨*D*⟩ and ARI (left axis) and mean classification error [%] (right axis); the vertical solid red line marks the default working-point subset, and the dashed red line marks the alternative working-point subset.

Interestingly, as sensors are progressively removed, interclass distances decrease monotonically, yet the classification error versus sensor count is not strictly monotonic (Figure 5A, right column). This fluctuation reflects cases where a retained sensor increases the overlap between Gaussian class distributions, partially offsetting the benefit of greater interclass separation. In this data set, Glc and Ure already form strongly overlapping Gaussians, so their separability is only weakly influenced by changes in distance to the far-removed Fol class. The default stopping criteria converged to a minimal two-sensor subset ((AC)_15_-SWCNTs and (AGGA)_7_-SWCNTs), which achieved the lowest classification error of 3.86%.

ACFSA V2.0 achieves improved separability by increasing the relative distance between the Gaussian centers of two closely spaced classes in the PC plane (Glc and Ure), at the expense of reducing their distance from the Fol class (see Figure 5A, left and middle columns, bottom subpanels), compared with the topology observed in the first iteration (Figure 5A, left and middle columns, top subpanels). This behavior arises by design: the wFS procedure prioritizes separation of classes that are initially close in the Gaussian representation, based on the rationale that clusters already far from the others are sufficiently separable. This result highlights a distinctive strength of ACFSA V2.0: although it is a supervised method, it provides interpretable insight into how specific sensor subsets modify class separability in the PC space, offering mechanistic transparency that can be valuable in data-analysis workflows.

For data set 2, we applied again the wFS activation of ACFSA V2.0. This data set proved challenging to classify, since, apart from the UA class, most classes’ Gaussians overlapped in the two-dimensional PC space (Figure 5B, left column subpanels, for both the initial and the working-point iterations). This has motivated the use of wFS for this data set. Moreover, the first two principal components explained only 66.8 and 15.6% of the total variance, respectively. Thus, restricting the analysis to two PCs may be limiting, whereas a three-dimensional implementation of ACFSA could potentially yield improved classification and will be tested in future work. As before, the QDA classifiers (Figure 5B, middle column) display distinctive decision boundaries, with the UA analyte being more dominant in the default working-point iteration (bottom panel).

For the minimal data set, two sensors were chosen at the default working point and three sensors at the alternative working point, with corresponding mean classification errors of 29.1 and 28.4% (see Figure 5C, right column). The ARI and mean intercluster distance are also reported. Overall, despite the overlapping Gaussians in PC space (Figure 5B left column), ACFSA V2.0 achieved a classifier that is able to distinguish LAC and UA from other classes reliably, as observed in Figure 5B middle column, bottom panel.

We reiterate that ACFSA V2.0 assumes per-class Gaussianity, which is particularly important here since each class had only *n*_*k*_ = 3 samples. To account for finite-sample uncertainty, we modified the covariance matrices of each class for data sets 1 and 2, as described in the Methods, thereby regularizing the fits and mitigating overfitting. Under this model, the inflated Gaussians define a parametric “test” distribution: by sampling from (or integrating over) these distributions, we estimated the scheme mean classification errors without an explicit train/test split. The covariance adjustment makes these estimates conservative and reliable.

### Advantages, Challenges, and Broader Context

ACFSA V2.0 is designed for small-sample sensor screening, where interpretability, transparency, and resistance to overfitting are essential, and the experimental measurement error for the examined analytes is predicted to have a Gaussian noise distribution. It provides a geometric, human-interpretable view in the PC1–PC2 plane, combining (i) class ellipses and decision maps, (ii) small-sample statistical corrections (eq 1), (iii) decision-aware feature elimination that emphasizes confusable class pairs (uFS and wFS), and (iv) an explicit cost–performance trade-off (eqs 2 and 3) for selecting compact yet accurate sensor subsets. Alongside the adjusted Rand index (ARI), ACFSA V2.0 tracks mean classification error and their evolution with sensor count, yielding an interpretable trace that can be directly compared to decision-map geometry to reveal the origins of classification performance.

These benefits come with scope constraints. Restricting modeling to the PC1–PC2 plane may overlook separability present in higher components, where a 3D or adaptive-dimensional extension would improve fidelity at added computational and memory cost (see Section S4 in the SI). Expanding the PCA representation based on cumulative explained variance remains an important direction for future work. While the present work considers only the transition between one and two dimensions, future extensions could incorporate additional principal components when the cumulative explained variance falls below a predefined threshold. Nevertheless, across the data sets examined here, the first two PCs capture a substantial fraction of the total variance and often explain an increasing share of the variance during feature elimination. In the default activation of ACFSA V2.0, feature selection is largely independent of the PCA embedding, since sensors are ranked by their individual χ^2^ discrimination power; when wFS is used, this independence is partially relaxed because the selection weights depend on class separability. As uninformative sensors are removed, the remaining subset increasingly reflects variance associated with separation between class means, which tends to concentrate variance in the leading PCs. Therefore, in practice, the PC1–PC2 representation was found to retain the dominant structure relevant for classification in the data sets examined here. A detailed discussion of this effect is provided in SI Section S9.

The Gaussian summary in PC space is a pragmatic approximation but may be challenged when sensor marginals are skewed or multimodal. Albeit using Ledoit-Wolf and ridge regularization for each class covariance, QDA accommodates unequal covariances yet can be sensitive when class ellipses are poorly conditioned or estimated from very few replicates, whereas Voronoi regains numerical stability at the expense of curved boundaries. Moreover, backward elimination is inherently greedy, although wFS partially mitigates this by emphasizing confusable class pairs, synergistic sensor combinations that minimize classification error can still be overlooked.

When simultaneous, same–event measurements across all sensors are not available, ACFSA V2.0 assembles a sample vector by concatenating sensor readings taken in separate runs. In this setting, ACFSA V2.0 necessarily treats the concatenated vector as if its entries were observed jointly. This assumption is defensible when sensor–specific measurement uncertainty dominates any event–level covariation across sensors. Otherwise, ACFSA V2.0 can still be beneficial, but to a reduced degree that depends on the relative importance of event–level cross–correlation compared with sensor measurement uncertainty and with the class–conditional summary statistics (means and variances).

The implications of concatenation depend on the acquisition regime. If sensors are event–correlated but recorded in separate runs, the true cross–sensor covariance induced by the shared event is not represented. This can bias the PCA geometry and class covariance estimates and, in turn, affect selection and classification. Conversely, if sensors are not event–correlated and are recorded in separate runs, concatenation can occasionally introduce weak, spurious dependencies due to small–sample variability. This effect typically diminishes as the number of replicates per analyte increases. In the best case scenario, event-correlated sensors are recorded simultaneously (each sample vector reflects the same event across sensors), so the induced covariance is present and naturally leveraged by ACFSA V2.0. Finally, if sensors are not event–correlated yet are recorded at the same time and entered jointly, no harm is expected, as any dependencies (or lack thereof) would emerge directly from the data rather than being imposed by concatenation.

Compared with other feature–selection schemes, ACFSA V2.0 is a backward–elimination wrapper tailored for screening data.^84^ Common classical alternatives include filters, embedded sparsity methods, heuristic searches such as genetic algorithms, and tree or ensemble–based importance.^85^ In this context, a wrapper serves as a pragmatic midpoint between computationally cheap, model-agnostic, accuracy-limited filters and costlier global searches with very high accuracy. It optimizes the sensor subset based on results from several iterations, utilizing a metaheuristic to eliminate sensors and achieve high accuracy. In the LOOCV benchmarking experiments conducted in this work, ACFSA V2.0 demonstrated competitive or improved performance relative to several widely used wrapper approaches for the examined data set. However, the relative advantage of ACFSA V2.0 compared with other wrapper methods across diverse data sets remains an important subject for future investigation.

Deep-learning feature–selection tends to excel when large labeled data sets are available and strong nonlinear interactions between features are present.^86^ However, with small sample sizes and few classes (typical of screening), flexible neural selectors can be prone to overfitting, as data are scarce. Recent benchmarks and surveys report that simpler classical filters/wrappers can match performance while retaining interpretability.^85^ Thus, given our small data set setting, a classical wrapper specialized for screening, ACFSA V2.0, offers an appropriate balance of accuracy, parsimony, and transparency.

## Conclusions

In this work, we have presented ACFSA V2.0, an interpretable and data-efficient workflow for optimizing cross-reactive sensor arrays under the small-sample conditions and Gaussian distribution that characterize most experimental screening campaigns. The central gap addressed by this framework is the lack of reliable and transparent sensor-selection tools that perform well when replicate counts are limited. Existing wrapper or filter methods often depend on large training sets or black-box classifier tuning, which can lead to unstable rankings and overfitting. By explicitly modeling measurement variability and class overlap, ACFSA V2.0 provides a principled route to sensor selection in settings where experimental constraints make data scarcity unavoidable.

Our framework introduces class-specific decision boundaries, variance-aware modeling for improved uncertainty calibration, and decision-aware feature scoring that favors sensors resolving the most confusable analytes. These additions preserve the intuitive geometric representation of low-dimensional decision maps while improving reliability when data are sparse. Across multiple independent data sets, ACFSA V2.0 consistently identified compact sensor subsets with low classification error and provided visual explanations that clarify why particular sensors were selected. Stress tests showed that variance inflation prevents overly optimistic performance estimates and that the method degrades predictably as analytes become harder to distinguish.

Benchmarking using a fully nested leave-one-out cross-validation (LOOCV) protocol further demonstrated that ACFSA V2.0 achieves competitive or improved classification accuracy compared with several widely used wrapper approaches, including SVM-RFE, RF-RFE, and PLS-DA-VIP-based elimination. In addition to predictive performance, the framework provides improved interpretability through its explicit low-dimensional decision maps and maintains substantially lower computational cost for the examined data set, highlighting the practical advantages of the approach for screening-type data sets.

Applications to additional experimental libraries^45,72^ demonstrate that the approach generalizes across sensing platforms, surface chemistries, and analyte families. This cross-system robustness highlights the importance of addressing the small-sample gap, since many chemical and biological sensing experiments cannot feasibly generate large data sets.

Future extensions may explore adaptive embeddings based on classification performance, automated PC-space dimensionality determination by cumulative explained variance, and hybrid or global subset-selection strategies. With an accompanying open-source implementation,^87^ ACFSA V2.0 offers an accessible and practical foundation for experimental screening and rational sensor-array design, providing compact and interpretable models that remain reliable in real-world, data-limited fingerprinting applications.

## Supplementary Material



## Data Availability

The data and software used to create this research are available upon request.

## Notes

Description of experimental data sets; ACFSA V2.0 algorithmic pipeline; statistical treatment of limited-sample covariance (inflation and regularization); computational complexity analysis; sensor-selection results on experimental and synthetic data sets; working-point sensitivity analysis; benchmarking against standard feature-selection methods; and validation of the two-dimensional PCA representation (PDF)
