# Supplementary material for: Rational Selection of Minimal Sensor Arrays for Analyte Fingerprinting
Source: Anal Chem. 2026 Apr 20;98(17):12390–403. doi: 10.1021/acs.analchem.5c07372 (PMC13150802; doi:10.1021/acs.analchem.5c07372)
Supplement: Supplementary file 1 [file ac5c07372_si_001.pdf]

Supporting Information

Rational Selection of Minimal Sensor Arrays for Analyte Fingerprinting

Michael Faran,[†] Gabriel Petresky,[†] Minyeong Yoon,[‡] Soo-Yeon Cho,[‡] and Gili Bisker[*,†,¶,§,‖,⊥,#]

†*School of Biomedical Engineering, Faculty of Engineering, Tel Aviv University, Tel Aviv 69978, Israel*

‡*School of Chemical Engineering, Sungkyunkwan University, Suwon 16419, Republic of Korea.*

¶*The Center for Physics and Chemistry of Living Systems, Tel Aviv University, Tel Aviv 6997801, Israel*

§*The Center for Nanoscience and Nanotechnology, Tel Aviv University, Tel Aviv 6997801, Israel*

‖*The Center for Light-Matter Interaction, Tel Aviv University, Tel Aviv 6997801, Israel*

⊥*The Center for Computational Molecular and Materials Science, Tel Aviv University, Tel Aviv 6997801, Israel*

#*Sagol School for Neuroscience, Tel Aviv University, Tel Aviv 6997801, Israel*

E-mail: bisker@tauex.tau.ac.il

Table of Contents

S1 Experimental Datasets Used in This Study **S3**

S2 Algorithmic Pipeline **S4**

S3 Limited-Sample Covariance Estimation: Inflation and Regularization **S9**

S4 AFSCA V2.0 Computational Time and Complexity **S17**

S5 ACFSA V2.0 Activation on the Default Dataset **S21**

S6 Working Point Sensitivity Test **S30**

S7 ACFSA V2.0 Benchmarking **S31**

S8 ACFSA V2.0 Activation on Dataset 1 and Dataset 2 **S41**

S9 Two-Dimensional PCA Representation Validity **S44**

References **S46**

S1 Experimental Datasets Used in This Study

The experimental datasets analyzed in this work were obtained from previously published nanosensor screening studies based on near-infrared fluorescent single-walled carbon nanotube (SWCNT) sensors. The datasets consist of fluorescence response matrices generated by exposing libraries of corona-functionalized SWCNT sensors to various analytes.

The default dataset corresponds to a library of 30 peptide-functionalized SWCNT sensors screened against five transition metal ions (Cu^{2+}, Ni^{2+}, Cr^{3+}, Pb^{2+}, and Hg^{2+}). The sensors were constructed by suspending HiPCO SWCNTs with Fmoc-peptide corona phases and measuring near-infrared fluorescence intensity changes upon analyte exposure. Detailed preparation procedures, peptide synthesis, SWCNT dispersion, UV oxidation, and fluorescence measurements are described in Ref.[1]

Dataset 1 consists of 20 DNA-functionalized SWCNT nanosensors designed for the detection of sweat-related analytes. In that study, diverse ssDNA sequences were used to generate distinct corona phases on SWCNTs, and fluorescence responses were recorded following analyte exposure under controlled experimental conditions. Full experimental details for nanosensor fabrication and screening procedures are provided in Ref.[2]

Dataset 2 comprises a library of 10 DNA–SWCNT nanosensors developed for the detection of urinary analytes using corona phase molecular recognition. The nanosensors were prepared by dispersing HiPCO SWCNTs with single-stranded DNA sequences to create analyte-sensitive corona phases, and fluorescence responses were recorded after incubation with the analytes. Detailed synthesis and characterization procedures are reported in Ref.[3]

In all cases, the datasets used here correspond to the normalized fluorescence responses measured for each sensor–analyte pair, forming the input matrices used by the ACFSA V2.0 analysis pipeline.

S2 Algorithmic Pipeline

This section presents the pseudocode for the ACFSA V2.0 algorithmic framework. Table S1 denotes the parameters used in the pseudocodes. Algorithm S1 is the main pseudocode, and its utility functions are Algorithms S2, S3, and S4. Algorithm S2 calculates the class Gaussian parameters, Algorithm S3 labels each sensor sample, and Algorithm S4 selects the eliminated sensor in the current ACFSA V2.0 iteration.

Table S1: **Notation used in Algorithm S1 and helpers**

**Symbol / Parameter**	**Description**
Main algorithm notation	
n	Total number of samples.
p	Total number of sensors (features).
$X \in \mathbb{R}^{n \times p}$	Input data matrix (samples $\times$ sensors).
K	Number of analyte classes.
$y \in \{1, \ldots, K\}^n$	Class labels (one per sample).
S	Active sensor subset.
Inflation	Finite-sample covariance/variance inflation toggle (`on/off`).
Classifier	Decision model: `QDA` or `Voronoi`.
FS	Feature-selection mode: `uFS` (uniform) or `wFS` (weighted).
$Z \in \mathbb{R}^{n \times 2}$	PCA scores in the PC1–PC2 plane.
$\{\mu_k, \Sigma_k\}$	Class-k Gaussian mean and covariance in PC space.
X_b	Per-sensor binned labels (SmartBinOneD output).
W_{ab}	Pair weight $= 1/\text{sep}_{ab}$ for classes a, b.
sep_{ab}	Distance between classes a and b zone centroids.
$\langle D \rangle$	Mean inter-class distance metric.
error, ARI	Mean classification error and Adjusted Rand Index traces.
Cumulative EV flag	Conditionally use only PC1 hyperparameter (0 or 1).
EV_1	Resulted PC1 explained-variance
τ_{EV}	PC1 explained-variance threshold hyperparameter; default 0.95
Helper algorithms notation	
Z_k	Rows of Z with $y_i{=}k$ (class-k scores).
n_k	Number of samples in class k.
$\mu_{(t)}$	Sorted class means (ascending) for a given sensor (1D).
$\sigma_{(t)}$	Sorted class means (ascending) standard deviations (1D).
b_t	Boundary between adjacent sorted classes t and $t{+}1$.
z_i	Zone label assigned to sample i by the sensor-specific ruler.
I_{ab}	Indices of samples with $y \in \{a, b\}$.
Score(s)	Feature score for sensor s (χ^2 or weighted pairwise χ^2).
Σ_{pool}	Pooled within-class covariance used as a shrinkage target (PC space).
v_k	Class-k 1D variance for a given sensor (before regularization).
v_{pool}	Pooled within-class variance for 1D binning.
α_k	Sample-size–dependent shrinkage weight ($\alpha_k = 1/n_k$).
γ_k	Ridge magnitude used to floor small eigenvalues in PC-space covariances.
$\lambda_{\min}(\Sigma_k)$	Smallest eigenvalue of the per-class covariance matrix

Algorithm S1: ACFSA V2.0: main loop

Input: $X \in \mathbb{R}^{n\times p}$, labels $y \in \{1,\ldots,K\}^n$, K, Inflation$\in$ {on, off}, Classifier$\in$ {QDA, Voronoi}, FS$\in$ {uFS, wFS}, CumulativeEVFlag $\in \{0,1\}$, $\tau_{\mathrm{EV}} \in (0,1)$ (PC1 explained-variance threshold; default 0.95);

Output: Traces of error/ARI/$\langle D\rangle$ vs. $|S|$; final S (size 1);

$S \leftarrow \{1,\ldots,p\}$;

Init traces;

while $|S| > 1$ **do**

  PCA on $X_{:,S}$; let EV_1 be the explained-variance fraction of PC1;

  if CumulativeEVFlag $= 1$ ***and*** $\mathrm{EV}_1 \geq \tau_{\mathrm{EV}}$ **then**

    scores $Z \leftarrow \mathrm{PC1} \in \mathbb{R}^{n\times 1}$ (1D mode);

  else

    scores $Z \leftarrow [\mathrm{PC1}, \mathrm{PC2}] \in \mathbb{R}^{n\times 2}$ (2D mode);

  $\{\mu_k, \Sigma_k\} \leftarrow$`ClassGaussians(`$Z, y, \mathit{Inflation}$`)`;

  model $\leftarrow$ **if** *Classifier* $=$ *QDA* **then**

    Build QDA from $\{\mu_k, \Sigma_k\}$;

  else

    Build Voronoi (nearest centroid) from $\{\mu_k\}$;

  Append metrics (error/ARI/$\langle D\rangle$) to traces;

  $X_b \leftarrow$`SmartBinOneD(`$X_{:,S}, y, K, \mathit{Classifier}, \mathit{Inflation}$`)`;

  `// Compute pair weights externally if wFS (no normalization):` $W_{ab} = 1/\mathrm{sep}_{ab}$ `from current model`

  $s^\star \leftarrow$`SelectFeature(`X_b, S, y, FS, K, W`)`;

  $S \leftarrow S \setminus \{s^\star\}$;

Algorithm S2: Helper: ClassGaussians (PC1-PC2 plane).

Input: Z (PC scores, $n \times 2$), y, Inflation$\in \{\text{on}, \text{off}\}$;

Output: $\{(\mu_k, \Sigma_k)\}_{k=1}^{K}$;

```
// Compute pooled within-class covariance target Σ_pool
```

$S_w \leftarrow 0_{2\times 2}$;

$n_{\text{tot}} \leftarrow 0$;

for $k = 1..K$ **do**

- $Z_k = \{Z_i : y_i = k\}$;
- $n_k = |Z_k|$;
- **if** $n_k < 2$ **then**
  - continue;
- $\mu_k = \text{mean}(Z_k)$;
- $\widetilde{Z}_k = Z_k - \mu_k$;
- $S_w \mathrel{+}= \widetilde{Z}_k^\top \widetilde{Z}_k$;
- $n_{\text{tot}} \mathrel{+}= n_k$;

$\Sigma_{\text{pool}} \leftarrow S_w / \max(n_{\text{tot}}, 1)$;

$\Sigma_{\text{pool}} \leftarrow \frac{1}{2}(\Sigma_{\text{pool}} + \Sigma_{\text{pool}}^\top)$;

for $k = 1..K$ **do**

- $Z_k = \{Z_i : y_i = k\}$;
- $n_k = |Z_k|$;
- $\mu_k = \text{mean}(Z_k)$;
- $\widetilde{Z}_k = Z_k - \mu_k$;
- $\Sigma_k = \text{cov}(\widetilde{Z}_k)$;
- **if** *Inflation* **then**
  - $\Sigma_k \leftarrow \text{Inflate}(\Sigma_k, n_k)$; `// finite-sample inflation`
- `// LW-style shrinkage toward pooled target (sample-size dependent)`
- $\alpha_k \leftarrow 1/n_k$; $\Sigma_k \leftarrow (1-\alpha_k)\Sigma_k + \alpha_k \Sigma_{\text{pool}}$;
- `// Eigenvalue-floor ridge for stable inversion (QDA)`
- $\bar{v} \leftarrow \text{tr}(\Sigma_{\text{pool}})/2$;
- $\tau_k \leftarrow \bar{v} / \max(n_k - 1, 1)$;
- $\gamma_k \leftarrow \max\big(0, \tau_k - \lambda_{\min}(\Sigma_k)\big)$;
- $\Sigma_k \leftarrow \Sigma_k + \gamma_k I$;
- $\Sigma_k \leftarrow \frac{1}{2}(\Sigma_k + \Sigma_k^\top)$;

Algorithm S3: Helper: SmartBinOneD (per-sensor K-zone labels).

Input: $X_{:,S}$ (samples×selected sensors), labels y, K, Boundary mode $\in \{\text{Voronoi}, \text{QDA}\}$, Inflation$\in \{\text{on}, \text{off}\}$;

Output: X_b (same size as $X_{:,S}$) with labels $\in \{1, \ldots, K\}$ per sensor (ascending by mean);

```
foreach sensor s ∈ S do
    // Estimate per-class 1D Gaussians for this sensor
    for k = 1..K do
        x_k ← {X_{i,s} : y_i = k};
        n_k ← |x_k|;
        μ_k ← mean(x_k);
        v_k ← var(x_k); if Inflation then
            v_k ← Inflate(v_k, n_k);
    // Pooled within-class variance target
    v_pool ← (Σ_{k=1}^{K} n_k v_k) / (Σ_{k=1}^{K} n_k);
    // Regularize per-class variances (no extra user knobs)
    for k = 1..K do
        v_k ← (1 − 1/n_k) v_k + (1/n_k) v_pool;
        v_k ← max(v_k, v_pool / max(n_k − 1, 1));
        σ_k ← √v_k;
    // Sort classes by mean; carry stds
    (μ_(1:K), σ_(1:K), ord) ← sort by μ_k ascending;
    // Compute K − 1 thresholds between adjacent sorted classes
    if Boundary = Voronoi then
        b_t ← ½(μ_(t) + μ_(t+1)) for t = 1..K − 1;
    else
        for t = 1..K − 1 do
            // 1D QDA boundary between N(μ_(t), σ²_(t)) and N(μ_(t+1), σ²_(t+1)), equal priors
            // Return the unique root inside (μ_(t), μ_(t+1)) if it exists; else midpoint
            b_t ← QdaBoundary1D(μ_(t), σ_(t), μ_(t+1), σ_(t+1));
    // Assign sample x to zone index z = 1 + #{t : x ≥ b_t} (gives labels 1..K in ascending-mean order)
    for i = 1..n do
        z_i ← 1 + Σ_{t=1}^{K−1} 1[X_{i,s} ≥ b_t];
        X_b(i, s) ← ord(z_i);
```

Algorithm S4: Helper: SelectFeature (uses pre-binned X_b).

Input: X_b (binned), S, y, FS$\in$ {uFS, wFS}, K, optional $W = \{W_{ab}\}$;

Output: $s^\star$ (sensor to remove);

```
if FS= uFS then
    foreach s ∈ S do
        build contingency table (bins 1..K) × (classes 1..K) from (X^binned_{:,s}, y);
        Score(s) ← χ²(X^binned_{:,s}, y);
else
    foreach s ∈ S do
        Score(s) ← 0;
        for 1 ≤ a < b ≤ K do
            I_ab = {i : y_i ∈ {a, b}};
            build (bins 1..K) × 2 contingency from (X^binned_{I_ab,s}, y_{I_ab});
            Score(s) += W_ab · χ²(X^binned_{I_ab,s}, y_{I_ab});      // W_ab = 1/sep_ab, no normalization
return s* = arg min_{s∈S} Score(s)                              // backward elim: drop lowest-scoring sensor
```

S3 Limited-Sample Covariance Estimation: Inflation and Regularization

Finite-Sample Predictive Variance Inflation. We set to analytically characterize variance inflation within each analyte class resulting from finite sample availability. This mitigates overfitting in Gaussian models, a potential pitfall in practical screening analyses with limited data.

Fix a class ($k \in \{1, \dots, K\}$) and one of its *local* PC axes (PC1 or PC2), obtained from the class-wise PCA used to draw the confidence ellipses in Figs. 3, 4, and 6 of the main text. Let the projected coordinates of the class samples along this chosen axis be $(x_1, \dots, x_n) \overset{\text{i.i.d.}}{\sim} \mathcal{N}(\mu, \sigma^2)$, with sample mean ($\bar{x} = \frac{1}{n}\sum_{i=1}^{n} x_i$), centered sum of squares $S = \sum_{i=1}^{n}(x_i - \bar{x})^2$, and unbiased variance $s^2 = S/(n-1)$. Because only a few replicates are typically available (n small), the mean and variance estimates carry substantial uncertainty. We therefore adopt a noninformative prior on (μ, σ^2).

Jeffreys' rule for a noninformative prior[4] can also be applied in its *joint* form, which

sets the prior proportional to the square root of the determinant of the Fisher information matrix:[5]

$$f(\mu, \sigma^2) \;\propto\; \sqrt{\det\, I(\mu, \sigma^2)}. \tag{S.1}$$

Calculating the the log-likelihood (μ, σ^2) Laplacian expectation value over the data distribution $f(x \mid \mu, \sigma^2)$, gives the Fisher information:

$$I(\mu, \sigma^2) \;=\; -\,\mathbb{E}_{x|\mu,\sigma^2}\big[\nabla^2_{(\mu,\sigma^2)}\, \ell(\mu, \sigma^2 \mid x)\big]. \tag{S.2}$$

Let $\theta = (\mu, \sigma^2)$ and $f(x \mid \theta)$ be the model density with log-likelihood $\ell(\theta \mid x) = \log f(x \mid \theta)$. Under standard regularity conditions (twice-differentiable ℓ, support not depending on θ, and interchange of integration and differentiation),

$$\mathbb{E}_{x|\theta}\big[\nabla_\theta \ell(\theta \mid x)\big] = \nabla_\theta \int f(x \mid \theta)\, dx = \nabla_\theta 1 = 0. \tag{S.3}$$

Differentiating w.r.t. θ and using the product rule,

$$\mathbf{0} = \nabla_\theta\, \mathbb{E}\big[\nabla_\theta \ell\big] = \mathbb{E}\big[\nabla^2_\theta \ell\big] + \mathbb{E}\big[\nabla_\theta \ell\, \nabla_\theta \ell^\top\big], \tag{S.4}$$

which yields the information equality

$$I(\theta) \;=\; -\mathbb{E}_{x|\theta}\big[\nabla^2_\theta \ell(\theta \mid x)\big] \;=\; \mathbb{E}_{x|\theta}\big[\nabla_\theta \ell(\theta \mid x)\, \nabla_\theta \ell(\theta \mid x)^\top\big]. \tag{S.5}$$

For $\theta = (\mu, \sigma^2)$, the Fisher information (by Eq. (S.5)) is the 2×2 matrix:

$$I(\mu, \sigma^2) = \begin{pmatrix} I_{\mu\mu} & I_{\mu,\sigma^2} \\ I_{\mu,\sigma^2} & I_{\sigma^2,\sigma^2} \end{pmatrix} = \begin{pmatrix} \mathbb{E}_{x|\theta}[(\partial_\mu \ell)^2] & \mathbb{E}_{x|\theta}[\partial_\mu \ell\, \partial_{\sigma^2} \ell] \\ \mathbb{E}_{x|\theta}[\partial_\mu \ell\, \partial_{\sigma^2} \ell] & \mathbb{E}_{x|\theta}[(\partial_{\sigma^2} \ell)^2] \end{pmatrix}, \tag{S.6}$$

whose determinant is calculated by:

$$\det I(\mu, \sigma^2) = I_{\mu\mu} \, I_{\sigma^2,\sigma^2} - \big(I_{\mu,\sigma^2}\big)^2. \tag{S.7}$$

For one observation $x \sim \mathcal{N}(\mu, \sigma^2)$, write $v = \sigma^2$. The derivatives of the log-likelihood are:

$$\partial_\mu \ell = \frac{x-\mu}{v}, \qquad \partial_v \ell = -\frac{1}{2v} + \frac{(x-\mu)^2}{2v^2}, \tag{S.8}$$

and taking expectations under $x \mid \mu, v$ (with $\mathbb{E}[x-\mu] = 0$, $\mathbb{E}[(x-\mu)^2] = v$) gives:

$$I_{\mu\mu} = \frac{1}{v}, \qquad I_{vv} = \frac{1}{2v^2}, \qquad I_{\mu v} = 0. \tag{S.9}$$

Thus, by Eq. (S.7),

$$\det I(\mu, v) = \frac{1}{v} \cdot \frac{1}{2v^2} - 0 = \frac{1}{2v^3}, \qquad \sqrt{\det I(\mu, v)} \propto v^{-3/2}, \tag{S.10}$$

which yields the joint Jeffreys prior in (μ, σ^2) (via Eq. (S.1)):

$$f(\mu, \sigma^2) \;\propto\; \sqrt{\det I(\mu, \sigma^2)} \;\propto\; (\sigma^2)^{-3/2} = \frac{1}{\sigma^3}. \tag{S.11}$$

For n i.i.d. samples, I scales by n and $\sqrt{\det I}$ by n, which is absorbed into the proportionality constant.

To derive the class-specific Gaussian inflation factor, we first obtain the posterior distributions of the mean and variance given the observed data. By Bayes' rule, the joint posterior is:

$$f(\mu, \sigma^2 \mid D) \;=\; \frac{f(D \mid \mu, \sigma^2)\, f(\mu, \sigma^2)}{f(D)}. \tag{S.12}$$

Hence $f(\mu, \sigma^2 \mid D) \propto f(D \mid \mu, \sigma^2)\, f(\mu, \sigma^2)$ is the joint posterior kernel. Under $x_i \overset{\text{i.i.d.}}{\sim}$

$\mathcal{N}(\mu, \sigma^2)$,

$$f(D \mid \mu, \sigma^2) = (2\pi\sigma^2)^{-n/2} \exp\Big(-\frac{1}{2\sigma^2}\sum_{i=1}^{n}(x_i - \mu)^2\Big). \tag{S.13}$$

Using $\sum_{i=1}^{n}(x_i - \mu)^2 = n(\mu - \bar{x})^2 + S$ and the prior (Eq. (S.11)), the joint posterior kernel becomes:

$$f(\mu, \sigma^2 \mid D) \;\propto\; (\sigma^2)^{-\frac{n}{2}-\frac{3}{2}} \exp\Big(-\frac{n(\mu - \bar{x})^2 + S}{2\sigma^2}\Big). \tag{S.14}$$

By Bayes' theorem for three variables,

$$f(\mu, \sigma^2 \mid D) \;=\; f(\mu \mid \sigma^2, D)\, f(\sigma^2 \mid D). \tag{S.15}$$

Dividing both sides of Eq. (S.15) by $f(\sigma^2 \mid D)$ and treating σ^2 as fixed, we obtain, up to a normalization constant independent of μ,

$$f(\mu \mid \sigma^2, D) \;\propto\; \exp\Big(-\frac{n}{2\sigma^2}(\mu - \bar{x})^2\Big) \quad\Longrightarrow\quad \mu \mid \sigma^2, D \sim \mathcal{N}\Big(\bar{x},\ \frac{\sigma^2}{n}\Big). \tag{S.16}$$

Integrating Eq. (S.14) over μ yields:

$$f(\sigma^2 \mid D) \;\propto\; (\sigma^2)^{-\frac{n+2}{2}} \exp\Big(-\frac{S}{2\sigma^2}\Big), \tag{S.17}$$

which is the inverse–χ^2 kernel[6] with $\nu = n$ and *ML* scale $s^2_{\mathrm{ML}} = S/n$:

$$\sigma^2 \mid D \sim \text{Inv-}\chi^2\big(\nu = n,\ s^2_{\mathrm{ML}} = S/n\big). \tag{S.18}$$

Equivalently, $\sigma^2 = \dfrac{\nu\, s^2_{\mathrm{ML}}}{Z} = \dfrac{S}{Z}$ with $Z \sim \chi^2_\nu = \chi^2_n$. Since $S = (n-1)\, s^2 = n\, s^2_{\mathrm{ML}}$, for $n > 2$, it follows that:

$$\mathbb{E}[\sigma^2 \mid D] = \frac{\nu\, s^2_{\mathrm{ML}}}{\nu - 2} = \frac{S}{n-2} = \frac{n-1}{n-2}\, s^2 \tag{S.19}$$

Moreover, by the law of total variance applied to μ w.r.t. the posterior of σ^2 and using

Eq. (S.16),

$$\operatorname{Var}(\mu \mid D) = \mathbb{E}_{\sigma^2|D}\big[\ \operatorname{Var}_\mu(\mu \mid \sigma^2, D)\ \big] \ + \ \operatorname{Var}_{\sigma^2|D}\big(\ \mathbb{E}_\mu(\mu \mid \sigma^2, D)\ \big). \tag{S.20}$$

Since $\mu \mid \sigma^2, D \sim \mathcal{N}\big(\bar{x},\ \sigma^2/n\big)$, we have $\operatorname{Var}_\mu(\mu \mid \sigma^2, D) = \sigma^2/n$ and $\mathbb{E}_\mu(\mu \mid \sigma^2, D) = \bar{x}$ (independent of σ^2). Thus the second term vanishes and

$$\operatorname{Var}(\mu \mid D) = \mathbb{E}_{\sigma^2|D}\Big[\frac{\sigma^2}{n}\Big] = \frac{1}{n}\,\mathbb{E}_{\sigma^2|D}[\sigma^2]. \tag{S.21}$$

For a new draw x_{new} from the class Gaussian, the conditional law of total variance gives:

$$\operatorname{Var}(x_{\text{new}} \mid D) = \mathbb{E}_{\mu,\sigma^2|D}\big[\ \operatorname{Var}(x_{\text{new}} \mid \mu, \sigma^2, D)\,\big] + \operatorname{Var}_{\mu,\sigma^2|D}\big(\,\mathbb{E}[x_{\text{new}} \mid \mu, \sigma^2, D]\,\big). \tag{S.22}$$

Given (μ, σ^2), $x_{\text{new}} \mid \mu, \sigma^2, D \sim \mathcal{N}(\mu, \sigma^2)$, so $\operatorname{Var}(x_{\text{new}} \mid \mu, \sigma^2, D) = \sigma^2$ and $\mathbb{E}[x_{\text{new}} \mid \mu, \sigma^2, D] = \mu$. Therefore,

$$\operatorname{Var}(x_{\text{new}} \mid D) = \mathbb{E}_{\sigma^2|D}[\sigma^2] \ + \ \operatorname{Var}_{\mu|D}(\mu) = \Big(1 + \frac{1}{n}\Big)\,\mathbb{E}_{\sigma^2|D}[\sigma^2], \tag{S.23}$$

and, using Eq. (S.19), for $n > 2$, we get the expression:

$$\boxed{\operatorname{Var}(x_{\text{new}} \mid D) = s^2\,\Big(1 + \frac{1}{n}\Big)\,\frac{n-1}{n-2}.} \tag{S.24}$$

Per-class, per-axis inflation and implementation. Writing n_k for the replicate count and s_k^2 for the unbiased sample variance in class k, we set the predictive variance on each PC axis to:

$$\tilde{\sigma}^2_{k,\text{pred}} = s_k^2\,\Big(1 + \frac{1}{n_k}\Big)\,\frac{n_k - 1}{n_k - 2} \qquad (n_k > 2), \tag{S.25}$$

and define a single inflation knob by:

$$\mathrm{STD\_buff}(n) \ = \ \sqrt{\left(1+\tfrac{1}{n}\right)\tfrac{n-1}{n-2}}, \qquad \tilde{\sigma}_k = s_k \cdot \mathrm{STD\_buff}(n_k). \tag{S.26}$$

In 2-D PC space, we apply the scalar factor $\mathrm{STD\_buff}(n_k)^2$ to the empirical class covariance (i.e., isotropic inflation of $\hat{\Sigma}_k$). This feature becomes particularly important when the number of samples per class differs, as class-specific inflation would alter the topology of the QDA decision boundaries. In this work, this effect is most pronounced in the LOOCV benchmarking tests described in the main text.

Shrinkage Toward the Pooled Within-Class Covariance. To regularize the class covariance estimates in the PC1–PC2 subspace, we shrink each empirical class covariance toward the pooled within-class covariance,[7] defined as Σ_{pool}:

$$\hat{\Sigma}_k = (1-\alpha_k)\,\Sigma_k + \alpha_k\,\Sigma_{\text{pool}}, \tag{S.27}$$

where Σ_{pool} is computed from the centered samples of each class as:

$$\Sigma_{\text{pool}} = \frac{1}{n_{\text{tot}}} \sum_{k=1}^{K} \sum_{i \in \mathcal{C}_k} (x_i - \bar{x}_k)(x_i - \bar{x}_k)^{\top}, \tag{S.28}$$

where $\mathcal{C}_k$ is the index set of samples belonging to class k, $\bar{x}_k$ is the mean of class k, and $n_{\text{tot}} = \sum_{k=1}^{K} n_k$ is the total number of samples across all classes.

The shrinkage weight α_k is estimated automatically using a Ledoit–Wolf–style procedure.[8,9] This step reduces small samples estimation noise while preserving the shared covariance structure across classes.

Eigenvalue Conditioning. After shrinkage, small eigenvalues are stabilized by adding a class-dependent ridge term.[10] Let $\hat{\Sigma}_k = Q_k \, \mathrm{diag}(\lambda_{k,i}) \, Q_k^{\top}$ be the eigendecomposition, where

$\lambda_{k,i}$ are the eigenvalues of class k, and Q_k is the orthonormal matrix of eigenvectors. We compute:

$$\tau_k = \frac{\mathrm{tr}(\Sigma_{\mathrm{pool}})}{p_{\mathrm{dim}}}\,\frac{1}{n_k - 1}, \qquad \gamma_k = \max\bigl(0, \tau_k - \lambda_{\min}(\hat{\Sigma}_k)\bigr), \tag{S.29}$$

and set:

$$\hat{\Sigma}_k \leftarrow \hat{\Sigma}_k + \gamma_k I. \tag{S.30}$$

This γ_k ridge regularization follows the classical ideas of regularized discriminant analysis and ridge regression,[10,11] with the ridge magnitude computed automatically: it is chosen so that the smallest eigenvalue is lifted to a noise floor determined by τ_k, preventing near-singular covariance estimates while minimally perturbing well-determined directions. The scale of this floor is set by the average variance $\mathrm{tr}(\Sigma_{\mathrm{pool}})/p_{\mathrm{dim}}$, and its dependence on $1/(n_k - 1)$ reflects the sampling variability of covariance estimates under finite samples.[8,9,12] Here $\lambda_{\min}(\hat{\Sigma}_k)$ denotes the smallest eigenvalue of $\hat{\Sigma}_k$; subtracting it from the target floor τ_k yields the minimal ridge γ_k required to raise that eigenvalue to τ_k, ensuring positive definiteness while leaving larger eigenvalues, and thus the main covariance structure, as unchanged as possible. Since this ridge regularization strengthens when the class sample size n_k is small, it ensures numerically stable covariance matrices and reliable QDA decision boundaries.

1-D QDA Binning for Feature Selection. Let v_k be the variance of class k computed from its n_k samples. We first compute the pooled within-class variance:

$$v_{\mathrm{pool}} = \frac{\sum_{k=1}^{K} n_k\, v_k}{\sum_{k=1}^{K} n_k}. \tag{S.31}$$

Each class variance is then updated according to:

$$v_k \;\leftarrow\; (1 - \alpha_k)\, v_k + \alpha_k\, v_{\mathrm{pool}}, \qquad \alpha_k = \frac{1}{n_k}, \tag{S.32}$$

followed by a variance-floor ridge regularization, using the same rationale as the 2D covariance conditioning performed earlier in the PCA space.:

$$v_k \leftarrow \max\left(v_k, \frac{v_{\text{pool}}}{n_k - 1}\right). \tag{S.33}$$

The resulting standard deviations $\sigma_k = \sqrt{v_k}$ are used to compute the 1D-QDA decision boundaries that define the bin thresholds.

S4 AFSCA V2.0 Computational Time and Complexity

We analyze the runtime of the default ACFSA V2.0 activation as a function of the number of samples n, total number of sensors p, number of classes K, and the current subset size s (which decreases from p to 1). Classification is performed in a reduced PCA space of fixed dimension $r = 2$. By definition, it follows that $n > K$, since each class possesses more than $n_k = 3$ samples in our framework. For error estimation, the PC space is discretized into a 2D grid (see per-class error estimation section in the main text), containing at least $\mathrm{Max}\{n, p\}$ grid points, R, along each principal component, i.e., $R > \mathrm{Max}\{n, p\}$, enabling accurate numerical evaluation of the overlap integrals.

Per-iteration costs. At each feature-elimination iteration (current subset size s), ACFSA V2.0 performs:

1. **Global PCA on the $n \times s$ matrix.**
   - A full singular value decomposition (SVD) is used and extracting components cost $\mathcal{O}\big(\mathrm{Min}\{n, s\}\,\mathrm{Max}\{n, s\}^2\big)$.[13]
2. **Per-class Gaussian fits in PC space (mean/covariance + finite-sample inflation).** Estimating $\{\mu_k, \Sigma_k\}_{k=1}^{K}$ in $\mathbb{R}^r$ costs $\mathcal{O}(nr)$ for the means calculation, and $\mathcal{O}(nr^2)$ for the covariances, where the latter is estimated by multiplication of two matrices.[14] Since $r = 2$ this cost is negligible relative to PCA when $s >> 2$.
3. **Classifier construction.**
   - *QDA (default):* accumulating covariances cost for all classes is $\mathcal{O}(Knr^2)$ and their inversion, required for the Gaussian summaries in the PC space, costs in $\mathcal{O}(Kr^3)$. With $r = 2$, this is constant-time per class and dominated by PCA. In the regularized-QDA variant, we additionally (i) compute a pooled covariance target in $\mathcal{O}(nr^2)$ per iteration, (ii) apply Ledoit–Wolf-style shrinkage[8] toward the

target and (iii) add a small ridge/eigenvalue floor for numerical stability;[10] these steps remain $\mathcal{O}(Knr^2)$ overall (and constant-time in practice for $r = 2$).

- *Voronoi (optional):* nearest-centroid with cost $\mathcal{O}(nr)$ for means; also negligible vs. PCA.

4. **Metric evaluation (error, ARI, $\langle D \rangle$).**

- *Interclass centroid distance $\langle D \rangle$.* Using Euclidean distances between the K class centroids, the pairwise distance matrix involves $\binom{K}{2}$ inner products, each costing $\mathcal{O}(r)$, for a total complexity of $\mathcal{O}(K^2 r)$.

- *Adjusted Rand Index (ARI).* Although the definition of ARI formally sums over all $\binom{n}{2}$ point pairs, in practice it is computed from the $K \times K$ contingency matrix between predicted and true labels. Constructing this matrix requires one pass over all n samples ($\mathcal{O}(n)$), and evaluating the ARI expression from these aggregated counts adds $\mathcal{O}(K^2)$.[15] The overall cost is therefore $\mathcal{O}(n + K^2)$.

- *Classification error.* For the empirical misclassification rate, QDA requires evaluating each of the K quadratic discriminants for all n samples ($\mathcal{O}(nKr^2)$), whereas the Voronoi (nearest-centroid) variant only computes Euclidean distances, scaling as $\mathcal{O}(nKr)$. In this work, the misclassification probability is estimated by numerical integration over a grid of $N_g = R^r$ points per class. Each grid point is assigned a class label according to the precomputed QDA or Voronoi decision boundaries, so the classification step requires no further discriminant evaluations. The overlap integral is then obtained by summing, over all grid cells, the product of each class's Gaussian kernel $N(\mu_k, \Sigma_k)$ with an indicator function that equals 1 when the grid cell shares the class label and 0 otherwise. The error is then calculated by 1 minus the overlap integral result. The computational cost of this procedure is therefore dominated by the evaluation of the K Gaussian kernels across the N_g grid points, scaling as $\mathcal{O}(KN_g r^2) = \mathcal{O}(KR^2 r^2)$, followed by a linear

$\mathcal{O}(KN_g)$ summation term that is negligible in comparison.

Overall, the metric evaluation stage therefore scales as $\mathcal{O}(K^2r) + \mathcal{O}(n + K^2) + \mathcal{O}(KR^2r^2) \approx \mathcal{O}(KR^2r^2)$ with the grid-based error term dominating for high-resolution integrations, and is comparable to or greater than the PCA cost.

5. **Decision-aware binning and feature scoring.** For each sensor $s' \in S$, we (i) compute $K-1$ thresholds from 1D class marginals and current model and (ii) build a contingency with K bins.

   - Thresholding and bin assignment across n values is $\mathcal{O}(nK)$ per sensor.
   - Pearson χ^2 scoring is $\mathcal{O}(K)$ per sensor.

   Hence, this step is $\mathcal{O}(nKs)$ per iteration; with small K which is below the PCA and error caclculuation costs.

Total cost across the elimination loop (per-iteration error). At iteration size s, the cost is:

$$T(s) = \underbrace{\mathcal{O}\big(\mathrm{Min}\{n,s\}\,\mathrm{Max}\{n,s\}^2\big)}_{\text{full SVD PCA}} + \underbrace{\mathcal{O}\big(K\,R^2r^2\big)}_{\text{grid error at } N_g=R^r}, \tag{S.34}$$

and this is summed from $s = p$ down to 1.

Case A: $n \geq p$**.** Here, $\min(n, s) = s$ and $\max(n, s) = n$, so the PCA term per iteration is $\mathcal{O}(ns^2)$. Summing yields the total time complexity as:

$$\sum_{s=1}^{p} T(s) = \boxed{\mathcal{O}(n\,p^3) + \mathcal{O}(K\,R^2r^2\,p)}. \tag{S.35}$$

Case B: $p \geq n$**.** Split the sum at $s = n$: for $s \geq n$, PCA is $\mathcal{O}(sn^2)$; for $s < n$, PCA is $\mathcal{O}(ns^2)$. Hence, the total time complexity is given by:

$$\sum_{s=1}^{p} T(s) \;=\; \underbrace{\mathcal{O}\Big(n^2 \sum_{s=n}^{p} s\Big)}_{=\;\mathcal{O}(n^2p^2) - \mathcal{O}(n^4)} \;+\; \underbrace{\mathcal{O}\Big(n \sum_{s=1}^{n-1} s^2\Big)}_{=\;\mathcal{O}(n^4)} \;+\; \underbrace{\mathcal{O}\big(K\,R^2 r^2\,p\big)}_{\text{grid error}} \tag{S.36}$$

$$= \boxed{\;\mathcal{O}(n^2p^2) \;+\; \mathcal{O}(n^4) \;+\; \mathcal{O}(K\,R^2 r^2\,p)\;}\,.$$

S5 ACFSA V2.0 Activation on the Default Dataset

Tables S2-S9 describe the representative metal-ion dataset (presented in the main text) data: sensor labels, indices, and ACFSA V2.0 activation elimination order for different scenarios. The sensors in these tables follow the format (m, n)-SWCNT-$AA_{\mathrm{OX/NOX}}$. Here, (m, n) are the chiral indices of the single-walled carbon nanotube (SWCNT), which specify its lattice structure (and thus diameter/chirality). "OX" denotes oxidized (acid-treated/carboxylated) SWCNTs, whereas "NOX" denotes non-oxidized (pristine) SWCNTs; protocols and rationale follow our previous work.[1] The AA tag (e.g., Arg, Glu, Lys, Cys, Gly) indicates the amino-acid corona used to disperse and functionalize the SWCNT. This nomenclature is consistent with our previous work.[1]

Table S2: Sensor numbers and labels for the metal-ion system, described in the main text.

Sensor Number	Sensor Label	Sensor Number	Sensor Label
1	$(6,5)$-SWCNT-Arg$_{\text{NOX}}$	16	$(7,5)$-SWCNT-Arg$_{\text{OX}}$
2	$(6,5)$-SWCNT-Glu$_{\text{NOX}}$	17	$(7,5)$-SWCNT-Glu$_{\text{OX}}$
3	$(6,5)$-SWCNT-Lys$_{\text{NOX}}$	18	$(7,5)$-SWCNT-Lys$_{\text{OX}}$
4	$(6,5)$-SWCNT-Cys$_{\text{NOX}}$	19	$(7,5)$-SWCNT-Cys$_{\text{OX}}$
5	$(6,5)$-SWCNT-Gly$_{\text{NOX}}$	20	$(7,5)$-SWCNT-Gly$_{\text{OX}}$
6	$(6,5)$-SWCNT-Arg$_{\text{OX}}$	21	$(9,4)$-SWCNT-Arg$_{\text{NOX}}$
7	$(6,5)$-SWCNT-Glu$_{\text{OX}}$	22	$(9,4)$-SWCNT-Glu$_{\text{NOX}}$
8	$(6,5)$-SWCNT-Lys$_{\text{OX}}$	23	$(9,4)$-SWCNT-Lys$_{\text{NOX}}$
9	$(6,5)$-SWCNT-Cys$_{\text{OX}}$	24	$(9,4)$-SWCNT-Cys$_{\text{NOX}}$
10	$(6,5)$-SWCNT-Gly$_{\text{OX}}$	25	$(9,4)$-SWCNT-Gly$_{\text{NOX}}$
11	$(7,5)$-SWCNT-Arg$_{\text{NOX}}$	26	$(9,4)$-SWCNT-Arg$_{\text{OX}}$
12	$(7,5)$-SWCNT-Glu$_{\text{NOX}}$	27	$(9,4)$-SWCNT-Glu$_{\text{OX}}$
13	$(7,5)$-SWCNT-Lys$_{\text{NOX}}$	28	$(9,4)$-SWCNT-Lys$_{\text{OX}}$
14	$(7,5)$-SWCNT-Cys$_{\text{NOX}}$	29	$(9,4)$-SWCNT-Cys$_{\text{OX}}$
15	$(7,5)$-SWCNT-Gly$_{\text{NOX}}$	30	$(9,4)$-SWCNT-Gly$_{\text{OX}}$

Table S3: Sensor elimination order for the metal-ion system in the default configuration, using the ACFSA V2.0.

Elimination Order	Sensor Label	Elimination Order	Sensor Label
1	$(7,5)$-SWCNT-Arg_{OX}	2	$(6,5)$-SWCNT-Arg_{OX}
3	$(6,5)$-SWCNT-Lys_{NOX}	4	$(9,4)$-SWCNT-Arg_{OX}
5	$(6,5)$-SWCNT-Arg_{NOX}	6	$(7,5)$-SWCNT-Lys_{OX}
7	$(9,4)$-SWCNT-Lys_{NOX}	8	$(7,5)$-SWCNT-Arg_{NOX}
9	$(6,5)$-SWCNT-Glu_{OX}	10	$(9,4)$-SWCNT-Arg_{NOX}
11	$(9,4)$-SWCNT-Cys_{NOX}	12	$(7,5)$-SWCNT-Lys_{NOX}
13	$(9,4)$-SWCNT-Lys_{OX}	14	$(9,4)$-SWCNT-Gly_{NOX}
15	$(7,5)$-SWCNT-Gly_{NOX}	16	$(6,5)$-SWCNT-Lys_{OX}
17	$(6,5)$-SWCNT-Cys_{NOX}	18	$(7,5)$-SWCNT-Cys_{NOX}
19	$(9,4)$-SWCNT-Cys_{OX}	20	$(9,4)$-SWCNT-Glu_{NOX}
21	$(7,5)$-SWCNT-Gly_{OX}	22	$(7,5)$-SWCNT-Cys_{OX}
23	$(9,4)$-SWCNT-Gly_{OX}	24	$(7,5)$-SWCNT-Glu_{OX}
25	$(7,5)$-SWCNT-Glu_{NOX}	26	$(6,5)$-SWCNT-Cys_{OX}
27	$(6,5)$-SWCNT-Glu_{NOX}	28	$(9,4)$-SWCNT-Glu_{OX}
29	$(6,5)$-SWCNT-Gly_{OX}	30	$(6,5)$-SWCNT-Gly_{NOX}

Table S4: Sensor elimination order for the metal-ion system with unadjusted non-inflated STD, using the ACFSA V2.0.

Elimination Order	Sensor Label	Elimination Order	Sensor Label
1	$(7,5)$-SWCNT-Arg_{OX}	2	$(6,5)$-SWCNT-Arg_{OX}
3	$(6,5)$-SWCNT-Lys_{NOX}	4	$(9,4)$-SWCNT-Arg_{OX}
5	$(7,5)$-SWCNT-Lys_{OX}	6	$(6,5)$-SWCNT-Arg_{NOX}
7	$(9,4)$-SWCNT-Cys_{NOX}	8	$(7,5)$-SWCNT-Arg_{NOX}
9	$(6,5)$-SWCNT-Glu_{OX}	10	$(9,4)$-SWCNT-Arg_{NOX}
11	$(9,4)$-SWCNT-Lys_{NOX}	12	$(7,5)$-SWCNT-Lys_{NOX}
13	$(9,4)$-SWCNT-Lys_{OX}	14	$(9,4)$-SWCNT-Gly_{NOX}
15	$(7,5)$-SWCNT-Gly_{NOX}	16	$(6,5)$-SWCNT-Lys_{OX}
17	$(6,5)$-SWCNT-Cys_{NOX}	18	$(9,4)$-SWCNT-Gly_{OX}
19	$(7,5)$-SWCNT-Cys_{NOX}	20	$(9,4)$-SWCNT-Cys_{OX}
21	$(9,4)$-SWCNT-Glu_{NOX}	22	$(7,5)$-SWCNT-Gly_{OX}
23	$(7,5)$-SWCNT-Cys_{OX}	24	$(7,5)$-SWCNT-Glu_{OX}
25	$(7,5)$-SWCNT-Glu_{NOX}	26	$(6,5)$-SWCNT-Cys_{OX}
27	$(6,5)$-SWCNT-Glu_{NOX}	28	$(9,4)$-SWCNT-Glu_{OX}
29	$(6,5)$-SWCNT-Gly_{OX}	30	$(6,5)$-SWCNT-Gly_{NOX}

Table S5: Sensor elimination order for the metal-ion system with Voronoi tesselation instead of QDA, using the ACFSA V2.0.

Elimination Order	Sensor Label	Elimination Order	Sensor Label
1	$(6,5)$-SWCNT-Arg$_{\mathrm{OX}}$	2	$(7,5)$-SWCNT-Arg$_{\mathrm{OX}}$
3	$(7,5)$-SWCNT-Arg$_{\mathrm{NOX}}$	4	$(9,4)$-SWCNT-Arg$_{\mathrm{OX}}$
5	$(7,5)$-SWCNT-Lys$_{\mathrm{OX}}$	6	$(6,5)$-SWCNT-Glu$_{\mathrm{OX}}$
7	$(6,5)$-SWCNT-Arg$_{\mathrm{NOX}}$	8	$(9,4)$-SWCNT-Lys$_{\mathrm{NOX}}$
9	$(9,4)$-SWCNT-Cys$_{\mathrm{NOX}}$	10	$(7,5)$-SWCNT-Lys$_{\mathrm{NOX}}$
11	$(9,4)$-SWCNT-Gly$_{\mathrm{NOX}}$	12	$(9,4)$-SWCNT-Arg$_{\mathrm{NOX}}$
13	$(6,5)$-SWCNT-Lys$_{\mathrm{NOX}}$	14	$(7,5)$-SWCNT-Gly$_{\mathrm{OX}}$
15	$(9,4)$-SWCNT-Glu$_{\mathrm{NOX}}$	16	$(7,5)$-SWCNT-Cys$_{\mathrm{OX}}$
17	$(7,5)$-SWCNT-Gly$_{\mathrm{NOX}}$	18	$(9,4)$-SWCNT-Cys$_{\mathrm{OX}}$
19	$(6,5)$-SWCNT-Lys$_{\mathrm{OX}}$	20	$(9,4)$-SWCNT-Lys$_{\mathrm{OX}}$
21	$(6,5)$-SWCNT-Cys$_{\mathrm{NOX}}$	22	$(7,5)$-SWCNT-Cys$_{\mathrm{NOX}}$
23	$(9,4)$-SWCNT-Gly$_{\mathrm{OX}}$	24	$(7,5)$-SWCNT-Glu$_{\mathrm{OX}}$
25	$(7,5)$-SWCNT-Glu$_{\mathrm{NOX}}$	26	$(6,5)$-SWCNT-Cys$_{\mathrm{OX}}$
27	$(6,5)$-SWCNT-Glu$_{\mathrm{NOX}}$	28	$(9,4)$-SWCNT-Glu$_{\mathrm{OX}}$
29	$(6,5)$-SWCNT-Gly$_{\mathrm{OX}}$	30	$(6,5)$-SWCNT-Gly$_{\mathrm{NOX}}$

Table S6: Sensor elimination order for the metal-ion system with weighted feature selection, using the ACFSA V2.0.

Elimination Order	Sensor Label	Elimination Order	Sensor Label
1	$(6,5)$-SWCNT-$\mathrm{Lys_{NOX}}$	2	$(6,5)$-SWCNT-$\mathrm{Glu_{OX}}$
3	$(6,5)$-SWCNT-$\mathrm{Arg_{OX}}$	4	$(7,5)$-SWCNT-$\mathrm{Arg_{OX}}$
5	$(9,4)$-SWCNT-$\mathrm{Arg_{OX}}$	6	$(7,5)$-SWCNT-$\mathrm{Arg_{NOX}}$
7	$(7,5)$-SWCNT-$\mathrm{Lys_{OX}}$	8	$(6,5)$-SWCNT-$\mathrm{Arg_{NOX}}$
9	$(9,4)$-SWCNT-$\mathrm{Arg_{NOX}}$	10	$(7,5)$-SWCNT-$\mathrm{Lys_{NOX}}$
11	$(9,4)$-SWCNT-$\mathrm{Lys_{OX}}$	12	$(9,4)$-SWCNT-$\mathrm{Gly_{NOX}}$
13	$(9,4)$-SWCNT-$\mathrm{Lys_{NOX}}$	14	$(9,4)$-SWCNT-$\mathrm{Cys_{NOX}}$
15	$(7,5)$-SWCNT-$\mathrm{Gly_{NOX}}$	16	$(9,4)$-SWCNT-$\mathrm{Cys_{OX}}$
17	$(7,5)$-SWCNT-$\mathrm{Gly_{OX}}$	18	$(7,5)$-SWCNT-$\mathrm{Cys_{OX}}$
19	$(7,5)$-SWCNT-$\mathrm{Cys_{NOX}}$	20	$(6,5)$-SWCNT-$\mathrm{Cys_{NOX}}$
21	$(9,4)$-SWCNT-$\mathrm{Glu_{NOX}}$	22	$(6,5)$-SWCNT-$\mathrm{Lys_{OX}}$
23	$(9,4)$-SWCNT-$\mathrm{Gly_{OX}}$	24	$(7,5)$-SWCNT-$\mathrm{Glu_{NOX}}$
25	$(7,5)$-SWCNT-$\mathrm{Glu_{OX}}$	26	$(6,5)$-SWCNT-$\mathrm{Glu_{NOX}}$
27	$(6,5)$-SWCNT-$\mathrm{Cys_{OX}}$	28	$(9,4)$-SWCNT-$\mathrm{Glu_{OX}}$
29	$(6,5)$-SWCNT-$\mathrm{Gly_{OX}}$	30	$(6,5)$-SWCNT-$\mathrm{Gly_{NOX}}$

Table S7: Sensor elimination order for the metal-ion system artificial dataset, (STD inflated by twofold), using the ACFSA V2.0.

Elimination Order	Sensor Label	Elimination Order	Sensor Label
1	$(6,5)$-SWCNT-Arg_{OX}	2	$(6,5)$-SWCNT-Lys_{NOX}
3	$(7,5)$-SWCNT-Arg_{OX}	4	$(9,4)$-SWCNT-Lys_{NOX}
5	$(7,5)$-SWCNT-Lys_{OX}	6	$(9,4)$-SWCNT-Arg_{OX}
7	$(6,5)$-SWCNT-Arg_{NOX}	8	$(6,5)$-SWCNT-Lys_{OX}
9	$(7,5)$-SWCNT-Arg_{NOX}	10	$(7,5)$-SWCNT-Cys_{NOX}
11	$(9,4)$-SWCNT-Lys_{OX}	12	$(9,4)$-SWCNT-Arg_{NOX}
13	$(7,5)$-SWCNT-Lys_{NOX}	14	$(6,5)$-SWCNT-Glu_{OX}
15	$(9,4)$-SWCNT-Gly_{NOX}	16	$(9,4)$-SWCNT-Cys_{NOX}
17	$(9,4)$-SWCNT-Glu_{NOX}	18	$(7,5)$-SWCNT-Glu_{OX}
19	$(6,5)$-SWCNT-Cys_{NOX}	20	$(9,4)$-SWCNT-Gly_{OX}
21	$(6,5)$-SWCNT-Glu_{NOX}	22	$(7,5)$-SWCNT-Cys_{OX}
23	$(9,4)$-SWCNT-Cys_{OX}	24	$(9,4)$-SWCNT-Glu_{OX}
25	$(7,5)$-SWCNT-Gly_{OX}	26	$(7,5)$-SWCNT-Gly_{NOX}
27	$(6,5)$-SWCNT-Gly_{OX}	28	$(7,5)$-SWCNT-Glu_{NOX}
29	$(6,5)$-SWCNT-Cys_{OX}	30	$(6,5)$-SWCNT-Gly_{NOX}

Table S8: Sensor elimination order for the metal-ion system artificial dataset, (STD inflated by fivefold), using the ACFSA V2.0.

Elimination Order	Sensor Label	Elimination Order	Sensor Label
1	$(6,5)$-SWCNT-Arg$_{\mathrm{OX}}$	2	$(7,5)$-SWCNT-Arg$_{\mathrm{OX}}$
3	$(6,5)$-SWCNT-Arg$_{\mathrm{NOX}}$	4	$(6,5)$-SWCNT-Lys$_{\mathrm{NOX}}$
5	$(9,4)$-SWCNT-Lys$_{\mathrm{NOX}}$	6	$(7,5)$-SWCNT-Lys$_{\mathrm{OX}}$
7	$(7,5)$-SWCNT-Lys$_{\mathrm{NOX}}$	8	$(6,5)$-SWCNT-Lys$_{\mathrm{OX}}$
9	$(9,4)$-SWCNT-Glu$_{\mathrm{NOX}}$	10	$(6,5)$-SWCNT-Glu$_{\mathrm{OX}}$
11	$(9,4)$-SWCNT-Arg$_{\mathrm{OX}}$	12	$(7,5)$-SWCNT-Cys$_{\mathrm{NOX}}$
13	$(9,4)$-SWCNT-Lys$_{\mathrm{OX}}$	14	$(9,4)$-SWCNT-Gly$_{\mathrm{NOX}}$
15	$(9,4)$-SWCNT-Gly$_{\mathrm{OX}}$	16	$(7,5)$-SWCNT-Glu$_{\mathrm{OX}}$
17	$(6,5)$-SWCNT-Cys$_{\mathrm{NOX}}$	18	$(7,5)$-SWCNT-Arg$_{\mathrm{NOX}}$
19	$(9,4)$-SWCNT-Arg$_{\mathrm{NOX}}$	20	$(7,5)$-SWCNT-Glu$_{\mathrm{NOX}}$
21	$(9,4)$-SWCNT-Glu$_{\mathrm{OX}}$	22	$(9,4)$-SWCNT-Cys$_{\mathrm{NOX}}$
23	$(6,5)$-SWCNT-Gly$_{\mathrm{NOX}}$	24	$(6,5)$-SWCNT-Glu$_{\mathrm{NOX}}$
25	$(6,5)$-SWCNT-Gly$_{\mathrm{OX}}$	26	$(7,5)$-SWCNT-Gly$_{\mathrm{NOX}}$
27	$(7,5)$-SWCNT-Cys$_{\mathrm{OX}}$	28	$(6,5)$-SWCNT-Cys$_{\mathrm{OX}}$
29	$(7,5)$-SWCNT-Gly$_{\mathrm{OX}}$	30	$(9,4)$-SWCNT-Cys$_{\mathrm{OX}}$

Table S9: Sensor elimination order for the metal-ion system artificial dataset, (STD inflated by tenfold), using the ACFSA V2.0.

Elimination Order	Sensor Label	Elimination Order	Sensor Label
1	$(6,5)$-SWCNT-Lys_{NOX}	2	$(6,5)$-SWCNT-Arg_{OX}
3	$(7,5)$-SWCNT-Lys_{OX}	4	$(7,5)$-SWCNT-Arg_{OX}
5	$(9,4)$-SWCNT-Glu_{NOX}	6	$(6,5)$-SWCNT-Arg_{NOX}
7	$(9,4)$-SWCNT-Lys_{NOX}	8	$(6,5)$-SWCNT-Lys_{OX}
9	$(7,5)$-SWCNT-Arg_{NOX}	10	$(9,4)$-SWCNT-Arg_{OX}
11	$(7,5)$-SWCNT-Lys_{NOX}	12	$(9,4)$-SWCNT-Gly_{NOX}
13	$(9,4)$-SWCNT-Gly_{OX}	14	$(7,5)$-SWCNT-Glu_{OX}
15	$(9,4)$-SWCNT-Arg_{NOX}	16	$(9,4)$-SWCNT-Lys_{OX}
17	$(6,5)$-SWCNT-Glu_{OX}	18	$(9,4)$-SWCNT-Glu_{OX}
19	$(6,5)$-SWCNT-Cys_{NOX}	20	$(7,5)$-SWCNT-Glu_{NOX}
21	$(7,5)$-SWCNT-Cys_{NOX}	22	$(6,5)$-SWCNT-Glu_{NOX}
23	$(7,5)$-SWCNT-Gly_{NOX}	24	$(6,5)$-SWCNT-Gly_{OX}
25	$(9,4)$-SWCNT-Cys_{NOX}	26	$(6,5)$-SWCNT-Gly_{NOX}
27	$(7,5)$-SWCNT-Gly_{OX}	28	$(9,4)$-SWCNT-Cys_{OX}
29	$(6,5)$-SWCNT-Cys_{OX}	30	$(7,5)$-SWCNT-Cys_{OX}

S6 Working Point Sensitivity Test

To assess the sensitivity of the operating point to the sensor addition penalty parameter η in Eqs. (2) and (3) of the main text, we computed the optimal number of selected sensors as a function of η (Fig. S1) based on the classifier error vs. sensor count presented in Fig. 3A of the main text. The results indicate that the relationship is piecewise constant, with two broad, stable regions in which the selected subset does not change, and a critical separation value of $\eta = 0.21$ separates them. The two values used in the main text, $\eta = 0.05$ and $\eta = 0.3$, lie in distinct regimes of this curve, corresponding to different stable sensor counts. This analysis indicates that the chosen working points represent qualitatively distinct trade-offs.

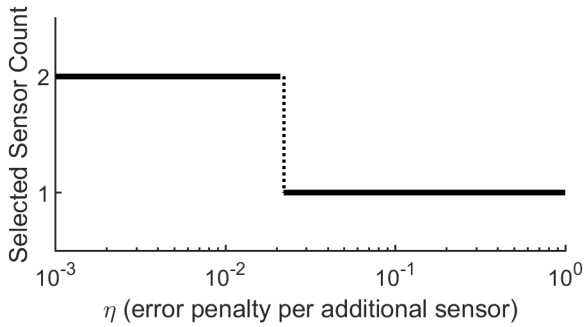

Figure S1: Sensitivity of the selected sensor subset to the penalty parameter η in Eq. (2). The optimal number of sensors, $l^*(\eta)$, is shown as a function of η on a logarithmic scale. The curve is piecewise constant, indicating broad stable regions where the selected subset does not change. The two working points used in the main text ($\eta = 0.05$ and $\eta = 0.3$) lie in distinct regimes of this curve, corresponding to different optimal sensor counts.

S7 ACFSA V2.0 Benchmarking

To evaluate the performance of ACFSA V2.0, we benchmarked the method against several widely used wrapper-based feature-selection approaches using a leave-one-out cross-validation (LOOCV) framework.[16] In LOOCV, one sample is left out for testing while the remaining samples are used for training; this process is repeated until each sample has served once as the test instance. Classification accuracy is then estimated as the fraction of correctly predicted samples across all LOOCV iterations. This empirical estimate differs from the analytical classification error derived earlier from the overlap between the decision regions and the Gaussian class distributions in the Voronoi or QDA map, which assumes that the underlying Gaussian class parameters are known without estimation uncertainty; consequently, the two quantities are expected to be similar but not identical. For each iteration, feature selection and classification were performed within the training set, ensuring that the evaluation remained fully nested and unbiased.

We compared ACFSA V2.0 with several established feature-selection strategies commonly used in chemometrics[17] and machine-learning pipelines, including recursive feature elimination with support vector machines (SVM-RFE), random-forest recursive feature elimination (RF-RFE), and partial least-squares variable importance in projection recursive elimination (PLS-DA-VIP-RFE).[18–20] In this section, we activate the cumulative explained variance flag, since we are interested in the average of many iterations in exchange for visual explainability. In the PLS-DA-VIP-RFE implementation used here, we adapted the conventional VIP-based filter procedure into an iterative elimination scheme. Rather than selecting all variables with VIP scores exceeding the commonly used threshold of 1, sensors were ranked according to their VIP values and removed sequentially within the training set. This procedure allowed construction of accuracy-versus-sensor curves comparable to those produced by ACFSA. Classification was performed using the standard PLS-DA model with linear decision boundaries in the latent-variable space. In addition, we included the original ACFSA V1.0 implementation as a reference baseline.

The benchmarking results for the original metal-ion dataset are shown in Fig. S2. Panel A reports the LOOCV classification accuracy as a function of the number of remaining sensors. For this dataset, RF-RFE, ACFSA V1.0, and ACFSA V2.0 achieve superior performance compared to the PLS-DA-VIP-RFE wrapper when the sensor subset is reduced to between 1 and 3 sensors. This regime is particularly relevant for practical applications where minimal sensor arrays are desired. The elimination order in these methods is determined by the feature importance measures provided by the respective classifiers: SVM-RFE ranks sensors according to the squared SVM weight coefficients, RF-RFE uses random-forest variable importance scores, and the PLS-DA-VIP-RFE approach ranks sensors according to their variable-importance-in-projection (VIP) scores. In all three cases, sensors are iteratively removed according to these rankings while the classifier is retrained at each step. The ACFSA framework constructs the classifier and feature-selection process jointly. The results indicate that, for this dataset, the ACFSA framework (ACFSA V1.0 and ACFSA V2.0) provides competitive or improved classification performance in the small-sensor regime, when compared to PLS-DA-VIP-RFE and SVM-RFE.

Runtime considerations also play an important role when evaluating feature-selection approaches. The total runtime required to perform the full sensor-elimination procedure, from the complete 30-sensor set down to a single sensor, was measured for each method using the LOOCV protocol. The results are summarized in Table S10. While RF-RFE achieves competitive classification accuracy, it requires substantially longer execution times compared with the other methods. In contrast, both ACFSA V1.0 and ACFSA V2.0 exhibit significantly lower computational cost while maintaining strong predictive performance. An additional advantage of the ACFSA framework lies in its interpretability. Because the classifier is constructed in a low-dimensional PCA space, the corresponding decision boundaries can be visualized directly in two dimensions. This allows inspection of how the removal of individual sensors influences class separation between consecutive elimination steps. By comparison, recursive feature elimination methods based on more complex machine-learning

models, such as RF-RFE, provide feature rankings but generally do not offer an equally direct geometric visualization of how feature removal alters the underlying decision boundary. When directly comparing the two ACFSA versions, the earlier Voronoi-based implementation slightly outperforms ACFSA V2.0 for sensor subsets containing five to six sensors in the original dataset.

It is important to emphasize that this benchmarking result should be interpreted as a *local comparison* rather than evidence of universal superiority. The performance of feature-selection algorithms can vary substantially across datasets with different statistical structures. For example, in the dataset examined here ACFSA achieved higher classification accuracy than the PLS-DA-VIP-RFE procedure used for comparison. In this implementation, the feature-elimination order was determined from VIP scores derived from a PLS-DA classifier with linear decision boundaries in the latent-variable space. Alternative classifier choices or elimination schemes could, in principle, yield different feature rankings and predictive performance on the same dataset. In principle, one could also envision workflows in which feature selection is performed in the original high-dimensional space using a chemometric model and the resulting subsets are subsequently projected onto a low-dimensional representation to aid visualization. While such approaches may offer similar interpretability benefits, a systematic evaluation of these alternatives across diverse datasets remains an interesting direction for future work. To further examine the relative behavior of ACFSA V2.0 beyond the original dataset, we therefore performed additional downsampling experiments using synthetic datasets generated from Gaussian distributions fitted to the original data, with the class standard deviations inflated tenfold to introduce increased variability.

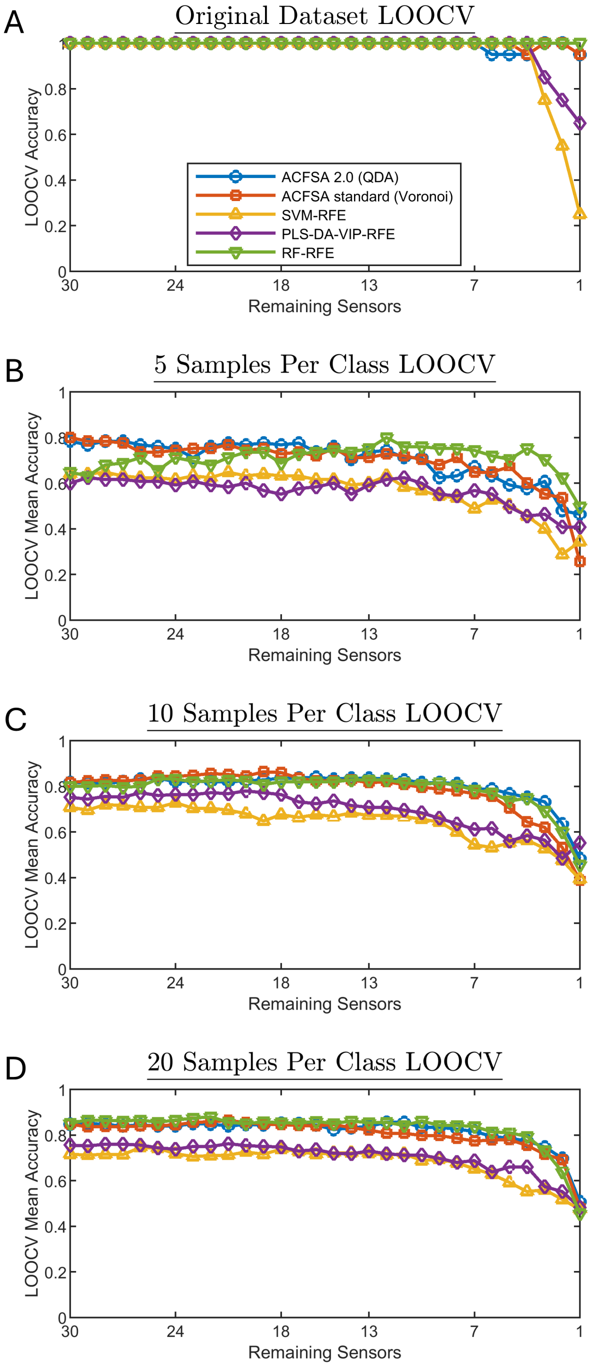

Figure S2: Benchmark comparison of ACFSA V2.0 with alternative feature-selection methods, including ACFSA V1.0. (A) LOOCV classification accuracy for the original metal-ion dataset as a function of the number of remaining sensors. (B–D) Results obtained on synthetic datasets generated from Gaussian distributions fitted to the original data, with the class standard deviations inflated tenfold to simulate increased variability. Different panels correspond to varying numbers of samples per class.

Table S10: Runtime of the different feature-selection methods for the original metal-ion dataset using LOOCV. Values are reported in seconds and minutes, where the runtime corresponds to the full feature-elimination procedure from 30 sensors down to a single remaining sensor.

Method	Runtime (s)	Runtime (min)
ACFSA 2.0 (QDA)	6.305	0.105
ACFSA standard (Voronoi)	2.305	0.038
SVM-RFE	23.369	0.389
PLS-DA-VIP-RFE	0.385	0.006
RF-RFE	270.697	4.512

Following the synthetic-data procedure described in Fig. 4C of the main text, we generated datasets with $n_k = 5$, 10, and 20 samples per class, with a tenfold increase in STD. For each dataset size, five independent samplings from the fitted class Gaussians were generated. For each sampling, sensor selection and classification were evaluated using the LOOCV protocol, and the resulting classification accuracy curves were averaged across the five realizations. The results are presented in Fig. S2B–D. The uncertainty across the five independent samplings for each iteration is reported in SI Tables S11-S13, corresponding to $n_k = 5$, 10, and 20 samples per class, respectively. The runtimes of the different methods for each dataset size are summarized in SI Table S14.

Across these synthetic datasets, RF-RFE again exhibits strong predictive performance but with a substantial increase in runtime relative to the ACFSA methods. In contrast, ACFSA V2.0 maintains competitive or improved accuracy relative to SVM-RFE and PLS-DA-VIP-RFE across all tested dataset sizes ($n_k = 5$, 10, and 20 samples per class), while remaining computationally efficient. Our initial expectation was that the ACFSA framework, designed to construct classifiers bottom-up under Gaussian assumptions and limited sample sizes, would primarily outperform machine-learning wrappers in the small-sample regime. Interestingly, the observed performance advantage persists even at $n_k = 20$ samples per

class.

We note, however, that this behavior likely reflects the approximately Gaussian structure of the metal-ion response distributions rather than a general superiority of the algorithm. Under different statistical conditions, other feature-selection strategies may perform better. A comprehensive evaluation across broader datasets therefore remains an important direction for future work.

Comparing ACFSA V2.0 with ACFSA V1.0 in the synthetic experiments reveals that the updated method generally produces a more favorable performance curve for $n_k = 10$ and $n_k = 20$ samples per class (Fig. S2C,D), particularly when the number of remaining sensors falls below six. In contrast, for the smallest dataset size ($n_k = 5$ samples per class), the earlier Voronoi-based implementation occasionally yields slightly better performance. This effect is likely due to increased uncertainty in covariance estimation when sample sizes are extremely limited, even when regularization is applied, which may favor the simpler Voronoi decision boundaries in this regime.

Table S11: LOOCV classification accuracy (mean ± std) across five synthetic samplings for the metal-ion system ($n_k = 5$ samples/class). Synthetic datasets were generated from class-specific Gaussian distributions fitted to the original dataset, with the standard deviation inflated tenfold. Sensor selection and classification were evaluated using LOOCV with internal cross-validation.

Remaining Sensors	ACFSA 2.0 (QDA)	ACFSA standard (Voronoi)	SVM-RFE	PLS-DA-VIP-RFE	RF-RFE
30	0.784±0.067	0.800±0.075	0.640±0.117	0.600±0.113	0.648±0.072
29	0.768±0.052	0.784±0.092	0.640±0.110	0.624±0.115	0.632±0.091
28	0.784±0.036	0.784±0.092	0.648±0.095	0.616±0.119	0.680±0.075
27	0.784±0.036	0.776±0.083	0.632±0.121	0.616±0.119	0.688±0.087
26	0.768±0.044	0.736±0.092	0.624±0.088	0.608±0.107	0.712±0.072
25	0.760±0.028	0.736±0.067	0.624±0.115	0.608±0.125	0.656±0.083
24	0.752±0.033	0.744±0.083	0.616±0.078	0.592±0.125	0.712±0.059
23	0.712±0.066	0.752±0.066	0.632±0.087	0.608±0.111	0.696±0.073
22	0.760±0.049	0.752±0.087	0.624±0.067	0.592±0.121	0.680±0.075
21	0.776±0.054	0.768±0.072	0.648±0.072	0.584±0.096	0.712±0.091
20	0.768±0.059	0.744±0.083	0.632±0.087	0.600±0.117	0.736±0.083
19	0.776±0.061	0.752±0.072	0.640±0.098	0.568±0.095	0.736±0.078
18	0.768±0.018	0.728±0.059	0.632±0.087	0.552±0.082	0.688±0.066
17	0.776±0.054	0.736±0.061	0.632±0.077	0.576±0.100	0.728±0.082
16	0.736±0.022	0.720±0.075	0.616±0.088	0.584±0.078	0.736±0.088
15	0.760±0.049	0.752±0.087	0.616±0.096	0.600±0.075	0.744±0.083
14	0.704±0.067	0.712±0.077	0.592±0.095	0.552±0.091	0.736±0.078
13	0.736±0.073	0.712±0.077	0.600±0.113	0.592±0.115	0.752±0.018
12	0.744±0.083	0.728±0.066	0.632±0.128	0.616±0.128	0.800±0.063
11	0.712±0.104	0.720±0.049	0.584±0.115	0.624±0.115	0.760±0.085
10	0.712±0.087	0.704±0.088	0.568±0.131	0.600±0.144	0.760±0.063
9	0.624±0.119	0.680±0.075	0.544±0.159	0.552±0.148	0.752±0.072
8	0.632±0.052	0.712±0.066	0.536±0.092	0.544±0.137	0.752±0.072
7	0.672±0.077	0.648±0.033	0.488±0.077	0.568±0.125	0.744±0.078
6	0.632±0.087	0.648±0.052	0.528±0.082	0.552±0.082	0.720±0.080
5	0.592±0.044	0.672±0.052	0.504±0.088	0.496±0.108	0.704±0.061
4	0.576±0.067	0.600±0.120	0.456±0.104	0.456±0.073	0.752±0.033
3	0.608±0.115	0.552±0.091	0.400±0.075	0.464±0.083	0.704±0.078
2	0.480±0.162	0.536±0.073	0.288±0.091	0.408±0.091	0.624±0.146
1	0.464±0.134	0.256±0.108	0.344±0.104	0.408±0.087	0.496±0.137

Table S12: LOOCV classification accuracy (mean ± std) across five synthetic samplings for the metal-ion system ($n_k = 10$ samples/class). Synthetic datasets were generated from class-specific Gaussian distributions fitted to the original dataset, with the standard deviation inflated tenfold. Sensor selection and classification were evaluated using LOOCV with internal cross-validation.

Remaining Sensors	ACFSA 2.0 (QDA)	ACFSA standard (Voronoi)	SVM-RFE	PLS-DA-VIP-RFE	RF-RFE
30	0.816±0.052	0.816±0.078	0.708±0.069	0.752±0.041	0.800±0.066
29	0.816±0.048	0.824±0.074	0.696±0.059	0.744±0.038	0.800±0.058
28	0.812±0.046	0.828±0.052	0.720±0.063	0.756±0.043	0.804±0.055
27	0.820±0.051	0.824±0.055	0.716±0.048	0.752±0.041	0.796±0.054
26	0.832±0.041	0.828±0.058	0.708±0.067	0.772±0.036	0.800±0.072
25	0.836±0.026	0.844±0.033	0.708±0.078	0.760±0.045	0.832±0.046
24	0.820±0.020	0.844±0.052	0.728±0.039	0.764±0.048	0.832±0.056
23	0.824±0.022	0.848±0.052	0.704±0.030	0.764±0.043	0.820±0.065
22	0.820±0.032	0.856±0.043	0.704±0.054	0.772±0.050	0.824±0.055
21	0.820±0.037	0.852±0.033	0.696±0.036	0.768±0.036	0.828±0.067
20	0.824±0.017	0.844±0.030	0.680±0.047	0.780±0.040	0.824±0.054
19	0.828±0.018	0.864±0.041	0.648±0.061	0.772±0.048	0.808±0.064
18	0.836±0.017	0.860±0.032	0.676±0.038	0.764±0.043	0.820±0.049
17	0.836±0.026	0.836±0.033	0.664±0.048	0.732±0.041	0.820±0.060
16	0.840±0.028	0.824±0.052	0.676±0.043	0.724±0.050	0.820±0.053
15	0.832±0.036	0.824±0.055	0.668±0.023	0.736±0.059	0.820±0.051
14	0.836±0.033	0.828±0.046	0.684±0.036	0.716±0.078	0.828±0.048
13	0.836±0.055	0.816±0.062	0.672±0.027	0.708±0.066	0.828±0.048
12	0.832±0.052	0.820±0.053	0.672±0.061	0.708±0.066	0.828±0.052
11	0.828±0.058	0.808±0.041	0.668±0.041	0.696±0.086	0.816±0.061
10	0.816±0.052	0.796±0.041	0.656±0.067	0.684±0.050	0.808±0.054
9	0.816±0.073	0.788±0.036	0.644±0.055	0.660±0.040	0.816±0.033
8	0.812±0.077	0.780±0.042	0.600±0.066	0.636±0.055	0.804±0.046
7	0.788±0.076	0.768±0.033	0.544±0.059	0.612±0.061	0.784±0.068
6	0.788±0.052	0.756±0.017	0.532±0.110	0.616±0.048	0.772±0.067
5	0.768±0.036	0.704±0.038	0.552±0.083	0.560±0.037	0.736±0.061
4	0.752±0.061	0.644±0.082	0.564±0.046	0.584±0.084	0.748±0.093
3	0.732±0.094	0.620±0.079	0.528±0.011	0.564±0.046	0.692±0.064
2	0.636±0.043	0.532±0.127	0.476±0.088	0.484±0.101	0.600±0.079
1	0.484±0.070	0.388±0.143	0.392±0.054	0.552±0.039	0.456±0.068

Table S13: LOOCV classification accuracy (mean ± std) across five synthetic samplings for the metal-ion system ($n_k = 20$ samples/class). Synthetic datasets were generated from class-specific Gaussian distributions fitted to the original dataset, with the standard deviation inflated tenfold. Sensor selection and classification were evaluated using LOOCV with internal cross-validation.

Remaining Sensors	ACFSA 2.0 (QDA)	ACFSA standard (Voronoi)	SVM-RFE	PLS-DA-VIP-RFE	RF-RFE
30	0.848±0.016	0.848±0.029	0.716±0.047	0.756±0.038	0.852±0.033
29	0.852±0.008	0.836±0.021	0.712±0.040	0.752±0.028	0.866±0.048
28	0.846±0.009	0.838±0.024	0.716±0.038	0.760±0.027	0.864±0.046
27	0.840±0.010	0.836±0.028	0.714±0.038	0.760±0.025	0.862±0.028
26	0.844±0.011	0.840±0.019	0.746±0.034	0.756±0.029	0.866±0.034
25	0.840±0.016	0.842±0.016	0.742±0.038	0.744±0.036	0.856±0.023
24	0.840±0.020	0.844±0.021	0.718±0.048	0.738±0.026	0.866±0.038
23	0.850±0.012	0.856±0.023	0.706±0.046	0.750±0.045	0.874±0.034
22	0.848±0.024	0.858±0.013	0.710±0.039	0.750±0.042	0.880±0.022
21	0.840±0.026	0.864±0.018	0.712±0.020	0.762±0.040	0.856±0.030
20	0.846±0.026	0.854±0.017	0.726±0.036	0.754±0.028	0.850±0.050
19	0.844±0.017	0.848±0.008	0.716±0.038	0.750±0.030	0.858±0.033
18	0.852±0.019	0.846±0.015	0.738±0.030	0.748±0.022	0.854±0.037
17	0.850±0.016	0.842±0.026	0.728±0.036	0.730±0.024	0.852±0.026
16	0.844±0.018	0.840±0.025	0.716±0.048	0.736±0.027	0.860±0.032
15	0.824±0.018	0.846±0.021	0.722±0.062	0.720±0.043	0.850±0.032
14	0.832±0.022	0.832±0.013	0.722±0.044	0.718±0.049	0.862±0.028
13	0.838±0.022	0.822±0.030	0.720±0.059	0.730±0.050	0.854±0.019
12	0.856±0.019	0.808±0.037	0.712±0.080	0.718±0.033	0.856±0.027
11	0.856±0.011	0.808±0.016	0.718±0.066	0.712±0.034	0.842±0.038
10	0.834±0.018	0.798±0.040	0.688±0.053	0.712±0.031	0.862±0.026
9	0.834±0.024	0.798±0.031	0.694±0.046	0.698±0.031	0.842±0.058
8	0.826±0.023	0.786±0.019	0.678±0.053	0.680±0.025	0.842±0.047
7	0.816±0.011	0.774±0.050	0.652±0.041	0.688±0.036	0.838±0.041
6	0.794±0.022	0.778±0.041	0.628±0.051	0.638±0.039	0.814±0.027
5	0.786±0.049	0.780±0.023	0.592±0.063	0.660±0.049	0.810±0.037
4	0.774±0.042	0.754±0.034	0.552±0.041	0.660±0.072	0.794±0.031
3	0.748±0.040	0.716±0.027	0.560±0.042	0.574±0.077	0.732±0.030
2	0.698±0.044	0.692±0.037	0.518±0.041	0.552±0.044	0.638±0.027
1	0.508±0.013	0.482±0.050	0.474±0.050	0.482±0.028	0.456±0.052

Table S14: Runtime of the different feature-selection methods for synthetic datasets evaluated using LOOCV, as the number of surviving sensors is reduced from the full set to a single sensor. Results are reported for different numbers of samples per class. Values are given as mean ± standard deviation (seconds) across five independent synthetic samplings, computed for the entire sensor-elimination procedure and averaged over the ensemble of cross-validation iterations.

Method	n_k samples/class	Runtime (s)
ACFSA 2.0 (QDA)	5	6.5517 ± 0.52146
ACFSA 2.0 (QDA)	10	9.6339 ± 0.79452
ACFSA 2.0 (QDA)	20	16.606 ± 1.7434
ACFSA standard (Voronoi)	5	2.4034 ± 0.10571
ACFSA standard (Voronoi)	10	5.1484 ± 0.46707
ACFSA standard (Voronoi)	20	10.59 ± 0.97414
PLS-DA-VIP-RFE	5	0.41661 ± 0.0090825
PLS-DA-VIP-RFE	10	0.80757 ± 0.037491
PLS-DA-VIP-RFE	20	1.8838 ± 0.16859
RF-RFE	5	332.67 ± 4.5258
RF-RFE	10	701.72 ± 36.181
RF-RFE	20	1624.1 ± 52.205
SVM-RFE	5	29.648 ± 1.0088
SVM-RFE	10	60.069 ± 3.1304
SVM-RFE	20	136.49 ± 8.6193

S8 ACFSA V2.0 Activation on Dataset 1 and Dataset 2

Figure S3 (S4) along tables S15 (S16) and S17 (S18) present additional data for dataset $1^2(2^3)$: Screening heat map, sensor numbers, labels, and elimination order. Sensors are represented as $(AC)_{15}$-SWCNTs, where the letters denote the single-stranded DNA motif adsorbed on the nanotube (A, C, G, T) and the subscript gives the number of motif repeats.

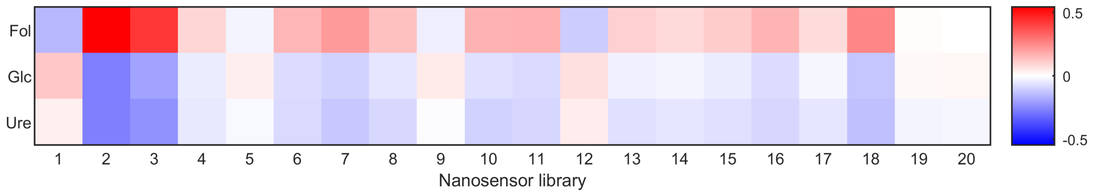

Figure S3: Screening heat map of nanosensor responses to meta-biome data, reproduced with permission from Lee et al.[2] Rows correspond to *Fol*, *Glc*, and *Ure* analytes; columns enumerate the nanosensor SWCNT library ($p = 20$). The corresponding sensor labels are listed in Table S15. Colors indicate the normalized response, highlighting class-dependent fingerprints across sensors.

Table S15: Sensor numbers and labels for dataset 1, reproduced with permission from Lee et al.[2]

Sensor Number	Sensor Label	Sensor Number	Sensor Label
1	$(AC)_{15}$-SWCNTs	11	$(CCCA)_7$-SWCNTs
2	$(ACA)_{10}$-SWCNTs	12	$(CGCA)_7$-SWCNTs
3	$(ACCA)_7$-SWCNTs	13	$(GAAC)_7$-SWCNTs
4	$(ACCG)_7$-SWCNTs	14	$(GACG)_7$-SWCNTs
5	$(ACG)_{10}$-SWCNTs	15	$(GAGC)_7$-SWCNTs
6	$(AG)_{15}$-SWCNTs	16	$(GCGA)_7$-SWCNTs
7	$(AGCA)_7$-SWCNTs	17	$(GGGC)_7$-SWCNTs
8	$(AGGA)_7$-SWCNTs	18	$(GT)_{15}$-SWCNTs
9	$(CACG)_7$-SWCNTs	19	$(GTTG)_7$-SWCNTs
10	$(CAGC)_7$-SWCNTs	20	$(TTTG)_7$-SWCNTs

Table S16: Sensor numbers and labels for dataset 2, reproduced with permission from Yoon et al.[3]

Sensor Number	Sensor Label	Sensor Number	Sensor Label
1	$(AAAC)_8$-SWCNTs	6	$(GCGA)_8$-SWCNTs
2	$(AAT)_{10}$-SWCNTs	7	$(GGGGT)_6$-SWCNTs
3	$(ACG)_{10}$-SWCNTs	8	$(GT)_{15}$-SWCNTs
4	$(GA)_{15}$-SWCNTs	9	$(TC)_{15}$-SWCNTs
5	$(GAAC)_8$-SWCNTs	10	$(TG)_{15}$-SWCNTs

Table S17: Sensor elimination order for dataset 1 with weighted feature selection, using the ACFSA V2.0.

Elimination Order	Sensor Label	Elimination Order	Sensor Label
1	$(TTTG)_7$-SWCNTs	2	$(CCCA)_7$-SWCNTs
3	$(AGCA)_7$-SWCNTs	4	$(AG)_{15}$-SWCNTs
5	$(ACCG)_7$-SWCNTs	6	$(ACA)_{10}$-SWCNTs
7	$(GTTG)_7$-SWCNTs	8	$(GT)_{15}$-SWCNTs
9	$(GAGC)_7$-SWCNTs	10	$(ACCA)_7$-SWCNTs
11	$(GAAC)_7$-SWCNTs	12	$(GGGC)_7$-SWCNTs
13	$(GCGA)_7$-SWCNTs	14	$(GACG)_7$-SWCNTs
15	$(CGCA)_7$-SWCNTs	16	$(CAGC)_7$-SWCNTs
17	$(ACG)_{10}$-SWCNTs	18	$(AGGA)_7$-SWCNTs
19	$(CACG)_7$-SWCNTs	20	$(AC)_{15}$-SWCNTs

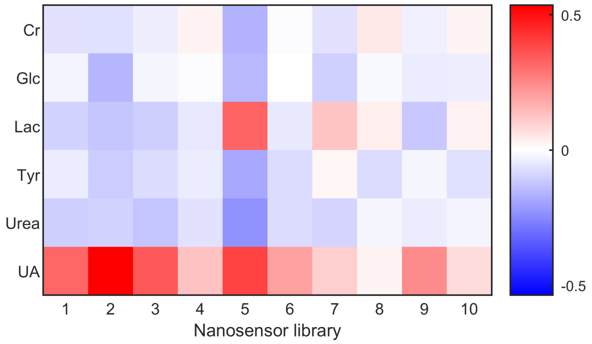

Figure S4: Screening heat map of nanosensor responses to meta-biome data, reproduced with permission from Yoon et al.[3] Rows correspond to *Cr*, *Glc*, *Lac*, *Tyr*, *Urea*, and *UA* analytes; columns enumerate the nanosensor SWCNT library ($p = 10$). The corresponding sensor labels are listed in Table S16. Colors indicate the normalized response, highlighting class-dependent fingerprints across sensors.

Table S18: Sensor elimination order for dataset 1 with weighted feature selection, using the ACFSA V2.0.

Elimination Order	Sensor Label	Elimination Order	Sensor Label
1	$(ACG)_{10}$-SWCNTs	2	$(TC)_{15}$-SWCNTs
3	$(AAT)_{10}$-SWCNTs	4	$(TG)_{15}$-SWCNTs
5	$(GCGA)_8$-SWCNTs	6	$(GGGGT)_6$-SWCNTs
7	$(GT)_{15}$-SWCNTs	8	$(GA)_{15}$-SWCNTs
9	$(GAAC)_8$-SWCNTs	10	$(AAAC)_8$-SWCNTs

S9 Two-Dimensional PCA Representation Validity

Across all simulated datasets examined in this work, the first two principal components capture a substantial fraction of the total variance. Specifically, the lowest combined explained variance (EV) observed is 48.5% (Fig. 4C of the main text), while in the remaining datasets the cumulative EV of PC1–PC2 exceeds 71%. These values indicate that the two-dimensional projection retains a significant portion of the structure present in the data.

More importantly, inspection of the PCA panels in Figs. 3-5 (left column) of the main text reveals a consistent trend: in later ACFSA iterations the first two principal components capture a progressively larger fraction of the total explained variance. This behavior arises naturally from the algorithmic design of ACFSA V2.0.

In the default activation of ACFSA V2.0, feature selection is performed using χ^2 tests applied independently to each sensor, requiring that each retained sensor individually supports class discrimination. Importantly, this uniform feature selection (FS) stage is intentionally decoupled from the PCA and classification steps. Sensors are therefore eliminated solely according to their individual discriminatory power, without regard to the geometry of the PCA embedding.

In representative chemometric datasets, samples belonging to the same analyte class are typically distributed around distinct class means, often approximated by Gaussian-like fluctuations. Under such conditions, informative sensors exhibit a clustered structure in which the within-class variance is smaller than the separation between class means. Consequently, the dominant source of variance in the data matrix arises from the separation between class centers, rather than from within-class fluctuations. When PCA is applied to such data, the leading principal components tend to align with directions that separate these clusters.

As the ACFSA iterations proceed and uninformative sensors are progressively removed, the remaining sensor subset increasingly consists of sensors that capture this class-separation signal. As a result, the variance associated with the separation between class means becomes more dominant relative to noise, and the first two principal components capture a larger

fraction of the total EV.

At the same time, the datasets considered in this work contain relatively few samples per class. In such small-sample regimes, covariance estimation is subject to finite-sample uncertainty, which may introduce spurious correlations and artificially inflate variance in certain directions. These effects can occasionally concentrate variance in specific principal components. However, the progressive removal of non-informative sensors reduces the dimensionality of noise contributions, thereby stabilizing the PCA representation in later iterations.

Taken together, these observations explain why the PC1–PC2 representation remains informative throughout the ACFSA elimination process. Because later iterations approach the minimal sensor configuration, which is the primary objective of the method, the use of a two-dimensional representation provides a practical compromise between dimensional fidelity and interpretability. The resulting PCA–QDA decision maps remain visually transparent while still capturing the dominant structure relevant for classification.

One potential limitation of selecting sensors individually is that discriminatory information arising from combinations of sensors could be overlooked. However, the final working point is selected by evaluating the full classification performance as a function of the number of retained sensors. Therefore, if an earlier iteration involving a larger sensor subset yields substantially improved classification performance, the cost function will favor that configuration. In this way, the algorithm balances the trade-off between sensor minimality and collective discriminatory power.

Finally, the weighted feature selection (wFS) variant partially reintroduces coupling between feature selection and the class-separation geometry. By emphasizing class pairs that are difficult to distinguish, wFS can help maintain separation between closely spaced classes, albeit at the cost of increased sensitivity to covariance-estimation noise.

References

(1) Petresky, G.; Faran, M.; Wulf, V.; Bisker, G. Metal-Ion Optical Fingerprinting Sensor Selection via an Analyte Classification and Feature Selection Algorithm. *Anal. Chem.* **2025**, *97*, 8821–8832.

(2) Lee, Y. S.; Shin, S.; Kang, G. R.; Lee, S.; Kim, D. W.; Park, S.; Cho, Y.; Lim, D.; Jeon, S. H.; Cho, S.-Y.; Pang, C. Spatiotemporal Molecular Tracing of Ultralow-Volume Biofluids via a Soft Skin-Adaptive Optical Monolithic Patch sensor. *Nat. Comm.* **2025**, *16*, 3272.

(3) Yoon, M.; Shin, S.; Lee, S.; Cho, S.-Y. Enzyme-Free Optical Detection of Uric Acid Using Corona Phase Molecular Recognition in Near-Infrared Fluorescent Single-Walled Carbon Nanotubes. *Nanoscale* **2025**, *17*, 10652–10662.

(4) Jeffreys, H. An Invariant Form for the Prior Probability in Estimation Problems. *Proc. R. Soc. London, Ser. A* **1946**, *186*, 453–461.

(5) Kay, S. M. *Fundamentals of Statistical Signal Processing, Volume I: Estimation Theory*; Prentice Hall, 1993.

(6) Gelman, A.; Carlin, J. B.; Stern, H. S.; Dunson, D. B.; Vehtari, A.; Rubin, D. B. *Bayesian Data Analysis*, 3rd ed.; CRC Press, 2013.

(7) Hastie, T.; Tibshirani, R.; Friedman, J. *The Elements of Statistical Learning*, 2nd ed.; Springer, 2009.

(8) Ledoit, O.; Wolf, M. A Well-Conditioned Estimator for Large-Dimensional Covariance Matrices. *J. Multivar. Anal.* **2004**, *88*, 365–411.

(9) Schäfer, J.; Strimmer, K. A Shrinkage Approach to Large-Scale Covariance Matrix Estimation and Implications for Functional Genomics. *Stat. Appl. Genet. Mol. Biol.* **2005**, *4*.

(10) Friedman, J. H. Regularized Discriminant Analysis. *J. Am. Stat. Assoc.* **1989**, *84*, 165–175.

(11) Hoerl, A. E.; Kennard, R. W. Ridge Regression: Biased Estimation for Nonorthogonal Problems. *Technometrics* **1970**, *12*, 55–67.

(12) Anderson, T. W. *An Introduction to Multivariate Statistical Analysis*; Wiley, 2003.

(13) Liang, F.; Shi, R.; Mo, Q. A Split-and-Merge Approach for Singular Value Decomposition of Large-Scale Matrices. *Stat. Inter.* **2016**, *9*, 453.

(14) Golub, G. H.; Van Loan, C. F. *Matrix computations*; JHU press, 2013.

(15) Rand, W. M. Objective Criteria for The Evaluation of Clustering Methods. *J. Amer. Stat. Assoc.* **1971**, *66*, 846–850.

(16) Stone, M. Cross-Validatory Choice and Assessment of Statistical Predictions. *J. R. Stat. Soc., Ser. B* **1974**, *36*, 111–147.

(17) Mehmood, T.; Liland, K. H.; Snipen, L.; Sæbø, S. A Review of Variable Selection Methods in Partial Least Squares Regression. *Chemom. Intell. Lab. Syst.* **2012**, *118*, 62–69.

(18) Guyon, I.; Weston, J.; Barnhill, S.; Vapnik, V. Gene Selection for Cancer Classification Using Support Vector Machines. *Mach. Learn.* **2002**, *46*, 389–422.

(19) Diaz-Uriarte, R.; De Andres, S. A. Gene Selection and Classification of Microarray Data Using Random Forest. *BMC Bioinform.* **2006**, *7*, 3.

(20) Chong, I.-G.; Jun, C.-H. Performance of Some Variable Selection Methods When Multicollinearity Is Present. *Chemom. Intell. Lab. Syst.* **2005**, *78*, 103–112.
